# Morphometric Analysis of *Coptotermes* spp. Soldier Caste (Blattodea: Rhinotermitidae) in Indonesia and Evidence of *Coptotermes gestroi* Extreme Head-Capsule Shapes

**DOI:** 10.3390/insects12050477

**Published:** 2021-05-20

**Authors:** Bramantyo Wikantyoso, Shu-Ping Tseng, Setiawan Khoirul Himmi, Sulaeman Yusuf, Tsuyoshi Yoshimura

**Affiliations:** 1 Research Institute for Sustainable Humanosphere (RISH), Kyoto University, Gokasho, Uji, Kyoto 611-0011, Japan; tsuyoshi@rish.kyoto-u.ac.jp; 2 Research Center for Biomaterials, Indonesian Institute of Sciences (LIPI) Jl. Raya Bogor km 46 Cibinong, Bogor 16911, Indonesia; khoirul_himmi@biomaterial.lipi.go.id (S.K.H.); sulaeman@biomaterial.lipi.go.id (S.Y.); 3 Department of Entomology, University of California, 900 University Avenue, Riverside, CA 92521, USA; shupingt@ucr.edu

**Keywords:** morphometry, geometric morphometrics, head capsule shape, postmentum waist, pronotum setae, *Coptotermes gestroi*, *Coptotermes curvignathus*, *Coptotermes elisae*

## Abstract

**Simple Summary:**

The morphological characteristics of the soldier caste in termites provide valuable taxonomic information at the species level. Head-shape variation in soldiers was often used as an indicative characteristic in some genera. While species with egg-shaped and waterdrop-shaped head capsule (HC), *Coptotermes gestroi* and *C. curvignathus*, respectively, are familiar in Indonesia, neither a measurement nor head index may avoid the subjectivity of shape interpretation. We conducted linear and geometric morphometrics analyses of soldiers’ HC of *Coptotermes* spp. obtained from various locations in Indonesia. Although subtle differences were observed, the posterior parts of the HC laterally expanded in a gradual manner in *C. gestroi, C. sepangensis,* and *C. curvignathus* in that order. Furthermore, three extreme head-shape variations of *C. gestroi* were found to exist in nature. Overall, we explicitly vocalized the complexity of utilizing HC morphometric measurement and shape for species determination in *Coptotermes* spp.

**Abstract:**

Linear and geometric morphometrics approaches were conducted to analyze the head capsule (HC) shape of collected soldier caste specimens of *Coptotermes* from various locations in Indonesia. The soldiers’ morphology was observed and measured. The results of the principal component analysis of the group of all species showed two important groups of variables, i.e., the body size and setae characteristics of the pronotum and head. The multicollinearity of the morphometric variables showed the importance of body measurements as well as important alternative characteristics such as the pronotum setae (PrS) and HC setae. Four trends of HC shape were observed across the species. Interestingly, three extreme shapes were depicted by geometric morphometrics of the *C. gestroi* HC. The phylogenetic tree inferred from 12S and 16S mitochondrial gene fragments showed high confidence for *C. gestroi* populations. The lateral expansion of the posterior part of the HC across the species was in accordance with the increasing of the number of hairlike setae on the pronotum and HC. These differences among species might be associated with mandible-force-related defensive labor and sensitivity to environmental stressors.

## 1. Introduction

Termites live in a variety of ecosystems and are widely distributed in tropical as well as subtropical regions, and low- and high-altitude montane forests, and they also infiltrate urbanized zones [1,2]. *Coptotermes* (Wasmann) were popularly related to anthropogenic settlement; they attack many kinds of commercial logs [3,4,5,6,7]. *Coptotermes gestroi* is known as an “urban pest termite” in tropical Southeast Asia; *C. elisae*, *C. curvignathus*, and *C. kalshoveni* are popular as “living trees pest termites” in Sumatra and Papua New Guinea’s plantation areas and dipterocarp forests; and *C. sepangensis* attacks suburban and rural areas in Malaysia [8,9,10,11,12,13,14]. Recently, *C. gestroi* behavior was observed attacking harvested and readily packed mangoes (*Mangifera indica* L.), which is unusual behavior for termites [15]. Despite their taxonomical complexity, all the aforementioned species are known to be well-distributed as termite pests in the realm of Malesiana.

The characteristics of termite soldiers’ sclerotized head capsule (hereinafter abbreviated to HC) provide valuable taxonomic information at the species level and have also helped to resolve the synonym issue [16,17,18]. However, it was reported that the HC size and shape were affected by the development of the frontal gland and muscle during soldier differentiation [19,20]. The variations of the head shape in soldiers were usually drawn dorsally or laterally and have been used as an indicative characteristic in some genera. For instance, an egg-shaped head is a familiar characteristic of *C. gestroi* [21], while a waterdrop-shaped head is known to be a characteristic of *C. curvignathus*, particularly in Indonesia. On the other hand, Kirton and Brown [22] were aware of the problems inherent in employing linear morphometric values in species delimitation. Hence, by collecting a considerable amount of HC morphometric data, they argued that both *C. havilandi* and *C. javanicus* were synonyms of *C. gestroi*. They also argued that *C. travians,* identified in several published studies [23,24,25,26,27], was a misidentification of *C. gestroi* due to the use of continuous variations. In *Odontotermes*, *Reticulitermes*, and *Heterotermes*, however, other characteristics’ variations such as setae positions and numbers were found to be important and specifically different [28,29,30,31,32].

Although several HC measurement records exist [9,16,25,33,34,35], neither a single measurement nor a head index has been used to represent shape. Hence, the difficulty in characterizing the HC shape has been a problem for some time. In practice, determining differences among shapes by visual evaluation is subjective, and so this criterion is difficult to apply [36]. While subjectivity cannot be completely eliminated, shape should be inferred in a specific way and should be based on the geometric shape, physical properties, or chemical composition corresponding to a biological function, environmental pressure, genes, or biomaterial fabrication [37,38].

In this study, the differences in the morphology of soldier caste of *Coptotermes* spp. from Indonesia were evaluated. We collected *Coptotermes* spp. from several sampling areas in Indonesia and analyzed the HC and other parts’ morphometrics using multivariate analysis [39,40,41]. Linear morphometric variations in *Coptotermes* spp., particularly *C. gestroi,* were exposed. We were the first to make use of geometric morphometrics of the soldiers’ HC and postmentum (hereinafter abbreviated to PS) in *Coptotermes* spp. We conducted a landmark-based analysis to elucidate the variation of shapes by focusing on a specific anatomical point on a Cartesian plane [42,43,44,45,46,47]. Since genetic information of *Coptotermes* spp. in Indonesia was limited [10,40,48,49], we inferred phylogenetic analysis from mitochondrial gene fragments to add important data for the genetic diversity.

## 2. Materials and Methods

### 2.1. Specimen Collection and Identification

Specimens were collected from nine locations in Indonesia from 2017–2019: around Sumatra Island (Simeulue Island, Karimunbesar Island, and Batam Island); Java Island from West Java province (Gunung Sindur, Cipinang, Cibinong, Karadenan, Parungpanjang) and the Special Capital Region of Jakarta (Pondok Kelapa); and Papua Island (Asmat Regency). Termites were preserved in 70% ethanol. Observations and photographs of the specimens were made using a digital microscope (VHX-5000, Keyence Corp., Osaka, Japan). Picture tracing was conducted using Drawing Pad (XP Pen Deco 2, Shenzhen, China) in Autodesk Sketchbook version 8.7.1. A total of 228 specimens of termite soldiers were measured (66 *C. curvignathus*, 10 *C. elisae*, 84 *C. gestroi*, 63 *C. sepangensis*, and 5 *C. kalshoveni* specimens). The identification was undertaken by collecting many characteristics from each specimen and matching them with references [16,22,23,24,25,26,50,51,52,53]. The Indonesian specimens were deposited at the Museum Zoologicum Bogoriense and Research Center for Biomaterials–Indonesian Institute of Sciences (LIPI), Cibinong, Indonesia. All Japan specimens belong to the Research Institute for Sustainable Humanosphere (RISH)–Kyoto University, Kyoto, Japan.

### 2.2. Linear Morphometric Analysis

**Figure 1:**
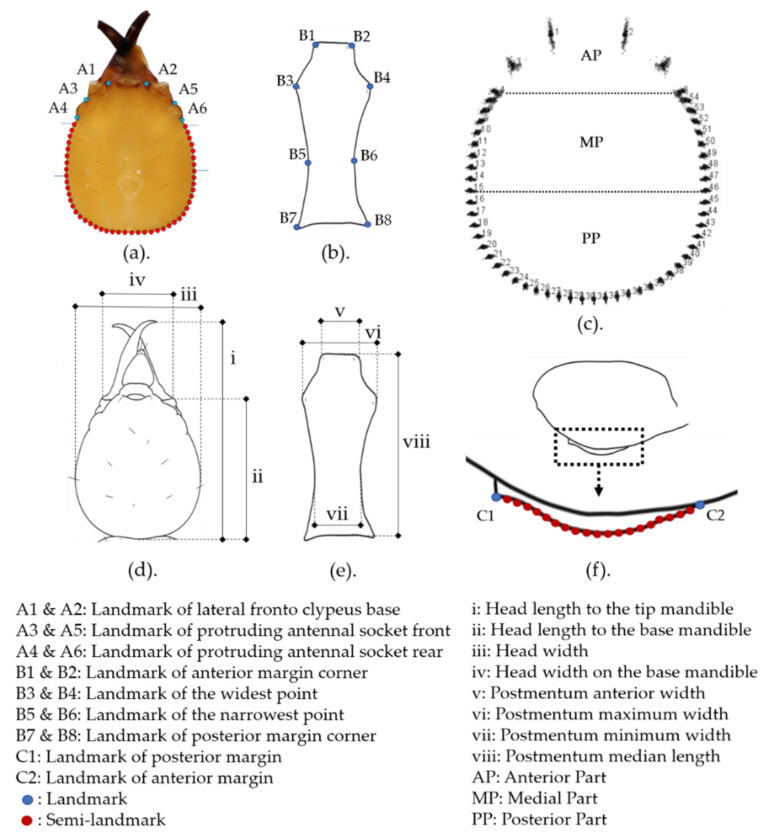
The landmark collection in: (**a**) head capsule; (**b**) ventral view postmentum; (**f**) lateral view postmentum. Linear morphometric measurement collection in: (**d**) head capsule; (**e**) postmentum. (**c**) The head capsule was randomly divided into three parts by an imaginary line on two pairs of landmarks (4 and 6, 15 and 46). Blue and red dots represent landmarks (6 in head capsule, 10 in postmentum) and semi-landmarks (48 in head capsule, 20 in postmentum), respectively. The distance between the semi-landmarks is equivalent to the increments of the curve length. Landmark determination in the postmentum follows the linear measurement guide. Roman numerals represent linear measurements.

Linear morphometric values were obtained from the distance between homologous points or landmarks of a set of particular landmarks (Figure 1) [54]. A total of 78 characteristics were collected in the initial observation of the specimens, and 34 of these were quantitative characteristics (Table S1) [30,55,56,57,58]. The characteristics were not subjected to the same statistical analysis if they were correlated with each other.

For example, the head width (HW) and head length to the base mandible (HLBM) characteristics will not be analyzed together with head index characteristics that consisted of the same constituents (HLBM/HW). This was done to avoid high collinearity in the exploratory analysis. All the data subjected to multivariate analysis were log10 transformed before being standardized to minimize skewness and non-normal distribution in some of the data due to allometry effect [59,60].

### 2.3. Geometric Morphometric Analysis

**Figure 2:**
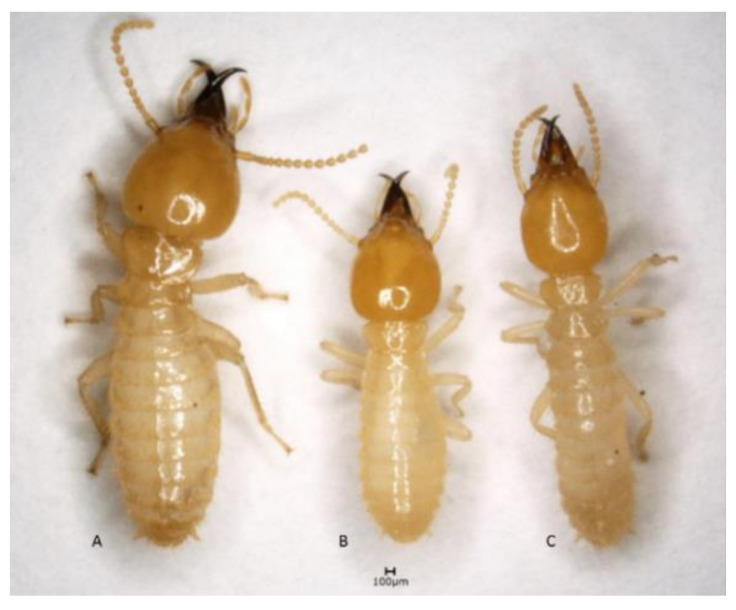
The two species, *C. curvignathus* (A) and *C. gestroi* (B,C) look different at first glance. Regardless of the body size, the head capsule (HC) shape of “B” looked as broad as “A” rather than that of “C”. Characteristics of HC shape alone would be unwieldy in the field practice.

The differences between the HC shapes of *C. curvignathus* and *C. gestroi* were subtle when judged by visual assessment (Figure 2), while the HC shape of *C. gestroi* specimens from Batam, Cipinang, and Gunung Sindur visually showed intriguing discrepancies to be further analyzed. It was obvious that the postmentum (hereinafter abbreviated to PS) shape varied depending on each species. Both HC and PS shape were further anatomized landmark-based approach. The PS waist images were taken from the ventral view, while HC images were taken from the dorsal view. The image collection was conducted by setting the specimens on a petri dish with cotton or Styrofoam soaked in 70% ethanol as a base. Two sets of images were collected using the digital microscope. All of the designated images were chosen and processed using image-processing software (tpsUtil version 1.78) [61]. A total of 210 soldiers were used to collect HC landmarks, 215 soldiers were used to collect PS ventral view landmarks, and 137 soldiers were used to collect PS lateral view landmarks.

Since there was no reference for determining landmarks in the termite HC and PS, landmarks and semi-landmarks were defined to represent the shape of the object and were determined from structures that were easily observed and repeatable. Landmarks are homolog coordinates that indicate a shape or biological form, while semi-landmarks are arbitrary coordinates along a curvature between landmarks [42,62,63]. In the PS, the eight landmarks chosen covered the anterior and posterior margins as well as the widest and narrowest PS width points. In the PS lateral view, two landmarks and 20 semi-landmarks were used on the curve. In the HC, six landmarks covering the third anterior part, and 48 semi-landmarks followed the curve of the head starting from the rear of the protruding antennal socket (Figure 1). The semi-landmarks were placed in the direction of the curve [64]. Landmarks and semi-landmarks were established and digitized using data acquisition software (tpsDig2 version 2.31) [65]. Scale factor determination followed the digital microscope scale reference. Full Procrustes-fit was executed to produce shape variables. The method of alignment by principal axis (the default method) was used in the analysis. The wireframe was created by connecting all the landmark centroids available after the superimposition process. The superimposition and collection of shape variables were performed using a software program for geometric morphometric analysis (MorphoJ version 1.07a) [44].

### 2.4. Statistical Analysis

Two sets of functional groups were created, i.e., one set for the group of all species and another one for the group of *C. gestroi*, based on the area of collection. The first set of functional groups was defined by three species, namely *C. curvignathus*, *C. gestroi*, and *C. sepangensis*. In a linear morphometric analysis, all *C. kalshoveni* were considered to be ungrouped cases due to the small number of samples collected in this study. The second set of functional groups consisted of *C. gestroi* specimens collected at each of seven locations. The functional groups were used in the analysis of linear and geometric morphometrics.

Principal component analysis (PCA) and discriminant functional analysis (DFA) of linear morphometric data and geometric morphometric data were conducted using the statistical packages IBM SPSS Statistics 27 (IBM Corp, Armonk, New York, NY, USA) and the MorphoJ program (version 1.07) [44], respectively. From 34 linear morphometric variables, 24 non-overlapped variables were utilized in PCA to find sets of further correlated characteristics (Table S2). A total of 54 landmarks in two dimensions were used as inputs in shape exploratory analysis.

The DFA was conducted to further polarize the three functional groups of all species and the seven functional locality-based groups of *C. gestroi* using the head morphology variation in specimens collected from seven locations (West Java province: 10 specimens from Cipinang, 10 from Gunung Sindur, 10 from Parung Panjang, 20 from Cibinong, and 10 from Karadenan; the Special Capital Region of Jakarta: 10 specimens from Pondok Kelapa; and Batam Island: 14 specimens). Collinearity diagnostic analysis was conducted to investigate the multicollinearity effect based on the PCA results. As many as 11 non-multicollinear characteristics (µm) were used in the initial canonical structure matrix to establish purposeful variables for further analysis of all of the species, while in the group of *C. gestroi*, 16 non-multicollinear characteristics were utilized in a stepwise fashion to independently select variables that would give the most contribution to the separation of the group [66,67]. In the stepwise DFA, Wilk’s lambda F value was used (default settings: 3.87 as the entry value; 2.71 as the removal value). Cross-validation was conducted for the results from both the grouping of all species and the *C. gestroi* grouping.

Statistical analysis of the centroid size and procrustes coordinates in geometric morphometric data was conducted in the MorphoJ program (version 1.07a) [44]. PCA was used to observe the shape variation in the procrustes coordinates. Canonical variate analysis (CVA) and DFA were applied to find the shape characteristics that best discriminate the data based on each functional group, respectively. The significance of the mahalanobis and procrustes distance between pairs of groups was assessed in 10,000 permutation runs. The total shape change was shown in transformation grids. To test the influence of size on the shape, regression analysis of each functional group was conducted in 10,000 permutation runs with procrustes distance and centroid size as data matrices [47,68]. Pool regression within group (*C. curvignathus*, *C. gestroi*, *C. sepangensis*, and *C. kalshoveni*) was applied to the PS shape’s procrustes distance. The differences in PS and HC shape within all species and within the group of *C. gestroi* were evaluated. The regression residuals value of procrustes coordinates was further utilized to perform grouping assessment. The level of significance in all statistical analysis in this study was *p* < 0.05.

### 2.5. Phylogenetic Analysis

The DNA extraction was carried out using one individual soldier of each species and one individual from each of the seven locations from which *C. gestroi* were collected (West Java province: Cipinang, Gunung Sindur, Cibinong, Karadenan, and Parungpanjang; the Special Capital Region of Jakarta: Pondok Kelapa; and Batam Island). The whole termite body was used and ground in a 1.5-mL vial to extract the total genomic DNA by following the protocol of the Gentra Puregene Tissue Kit (Qiagen GmbH, Hilden, Germany). Two pairs of mitochondrial primers were used for targeting the 12S and 16S genes. Amplification of the 12S gene was performed by 12SF (5′-TACTATGTTACGACTTAT-3′) and 12SR (5′-AAACTAGGATTAGATACCC-3′) [40,69], while 16SF1 (modified) (5′-GATWACGCTGTTATCCCTAAG-3′) and 16SR-N/A-03 (designed in the present study) (5′-GTTAAGATTAAGGGGGACTAG-3′) were used to amplify the 16S gene.

The polymerase chain reaction cycles were as follows: precycle denaturation (94 °C–3 min); 40 cycles of denaturation (94 °C–30 s), annealing (45 °C for 12 s and 50 °C for 16 s–45 s), and extension (72 °C–1 min); post-cycle extension (72 °C–7 min). The amplification results were purified according to the Fastgene purification kit protocol and sequenced by DNA Sequencing Core, Kyoto University, Kyoto, Japan, and Eurofins DNA Sequence, Tokyo, Japan.

The sequence results were manually edited and combined by using a biological sequence alignment editor (BioEdit version 7.0.5.3) [70] before the analysis was performed. The multiple alignments of several consensus sequences were performed using a multiple alignment program (ClustalX 2.1) [71]. *Odontetermes* sp. was used as an outgroup in the analysis. The other sequences of *Coptotermes* spp. included in the study were recruited from GenBank (www.ncbi.nlm.nih.gov (accessed on 28 May 2020)) (Table 1). The genetic distance was analyzed according to the Kimura two-parameter model of sequence evolution in Mega 5.2 [72,73]. The substitution model was set using a nucleotide substitution model selection script (Kakusan 3.0) [74]. Phylogeny analysis was conducted by the Bayesian inference method using MrBayes 3.2.7a [75] and by the maximum likelihood method using Mega 5.2 [76]. A maximum likelihood test was done with 10,000 bootstrap replications, and the GTR+G substitution model was used. Bayesian N-generation was 10 × 10^6^ with 3 × 10^5^ burn-in, and the substitution model was GTR_G (the number of rate categories of gamma distribution was set to eight, and the state frequencies were set to [1]) for each partitioned gene. The tree was edited in a graphical viewer (Figure Tree 1.4.3). The confidence level was labeled at the nodes. Bootstrap levels equal to 70% or higher were considered to show the true clade [77]. When using MrBayes, a 95% probability or higher was considered significant [78,79,80].

## 3. Results

### 3.1. Soldier Morphology Characteristics of Coptotermes (Wasmann)

**Figure 3:**
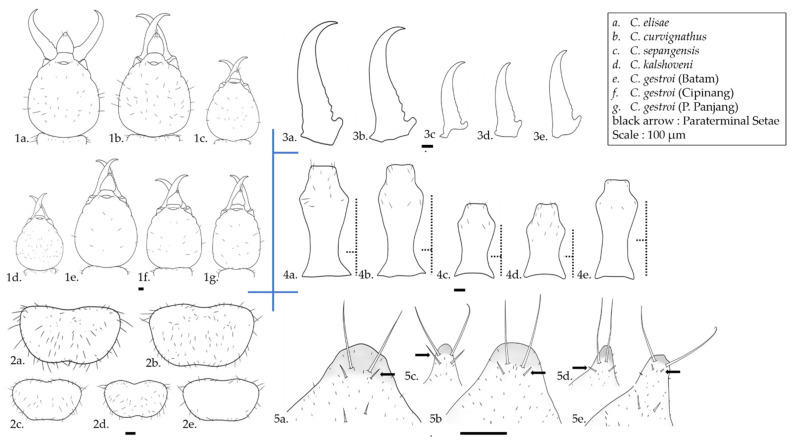
Comparison of *Coptotermes* spp. ((1). Head capsule, (2). Pronotum, (3). Left mandible curvature, (4). Postmentum, (5). Labrum paraterminal setae). Tracing was conducted in Autodesk Sketchbook version 8.7.1. Scale in 100 µm.

**Figure 4:**
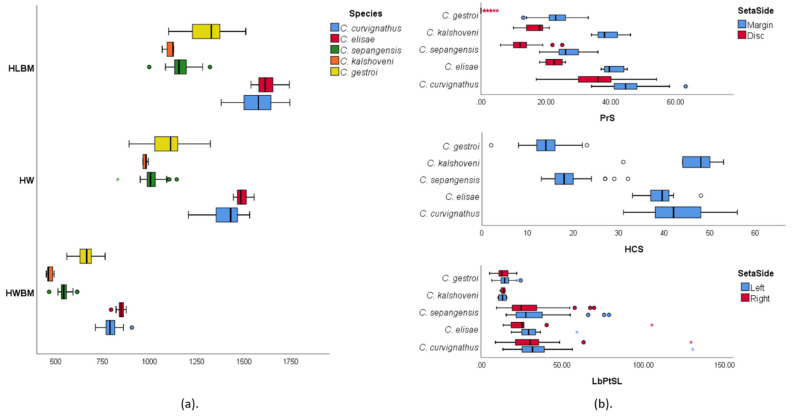
Multicollinearity showed shifting in a linear fashion of one variable in accordance with the others: (**a**) Multicollinearity of some variables that are related to the head capsule in clade X of the principal component analysis (PCA), namely the head length to base mandible (HLBM), head width (HW), and head width on the base mandible (HWBM); (**b**) Fewer collinear variables in clade Y of the PCA, namely the pronotum setae (PrS) and head capsule setae (HCS) including the labrum paraterminal setae length (LbPtSL) of the group of all species. The species (A) and setae positioning (B) are represented in different colors.

In this analysis, 44 qualitative characteristics and 34 quantitative data were collected (Tables S1 and S3). The morphology of each species is illustrated in Figure 3. *Coptotermes curvignathus* was the largest species observed in this study. It generally had a subangular-shaped and posteriorly bulged HC with a considerable number of setae on the HC (31–56) and pronotum (61–113 setae). Generally, one pair of setae was observed around the fontanelle. However, the setae varied in number from 1–4. At the tip of the labrum, there was one pair of long terminal setae and one pair of medium-length para-terminal setae (2+2M labrum seta formula) (Figure 4b). The mandibles were strongly incurved. Ventrally, a low waist PS was observed with diverse numbers of setae ranging up to 18 at the anterior edge.

Although the number of *C. elisae* samples was very limited (n = 10), the body measurements of *C. elisae* overlapped with those of *C. curvignathus*, the sample number of which was much higher in this study. However, the shape of the head was slightly distinguished from that of *C. curvignathus*. We found that it was difficult to distinguish *C. elisae* from *C. curvignathus* by solely looking into its soldier morphology without conducting a genetic analysis.

The characteristics of *C. gestroi* were the most varied scale data we observed in this study. Three different HC shapes were observed: (1) Several specimens had broadly oval head-shape, regardless of their size—the head looked similar to the head of *C. curvignathus* from the dorsal view at first glance; (2) few specimens had subangular head-shape (Figure 2); while (3) majority specimens had the usual egg-shape. The fontanelle was generally decorated by two setae. A variation occurred in which a few individuals in some studied populations had 3–4 setae around the fontanelle. The labrum at the terminal site had one pair of setae and one pair of short para-terminal setae (2+2S labrum seta formula) (Figure 4b). In some specimens, the existence of para-terminal setae was either not obvious or did not exist. The mandibles were slightly incurved, rather straight. The pronotum was flat with setae mostly found at the edge of the pronotum. Few or no setae were observed on the disc part of the pronotum (0–5 setae). Ventrally, the middle waist PS was observed across the samples.

The HC shape of *C. sepangensis* was generally narrow at the anterior part and rounded at the posterior part. A moderate number of setae decorated the HC (13–32 setae). Generally, there were two setae at the fontanelle. A few individuals in the study showed three setae around the fontanelle and it showed 2+2M labrum seta formula (Figure 4b). The mandibles were quite incurved with curvature starting at the early part of the mandible. The level of curvature varied among specimens. The lateral view showed that the PS bulged ventrally. From the ventral view, a low-waist PS was observed.

The HC shape of *C. kalshoveni* was generally broad at the medial part with a sudden narrowing at the anterior part just behind the mandible. A massive number of setae was observed on the HC (31–53 setae). Two pairs of setae were situated around the fontanelle. It showed 2+2S labrum seta formula (Figure 4b). The mandibles were less incurved, rather straight, with the curvature starting at the final third of the distal part. Laterally, the PS bulged ventrally. From the ventral view, a low-waist PS with a high number of setae (11–13 setae) at the anterior part was observed. In the group of *Coptotermes* spp. collected in this study, *C. kalshoveni* was the only species with a waist width wider than the anterior margin width. The pronotum was flat and had a high number of setae scattered on the pronotum disc and marginal side (46–64 setae).

### 3.2. Linear Morphometrics

High pair-wise correlations were observed within 24 of the analyzed data (Figure 4). As many as 14 characteristics had a pair-wise correlation coefficient of more than 0.90. (Table S1). Diagnostics were conducted to confirm the collinearity of the characteristics by regression linear analysis of highly correlated variables. Since we wanted to use the HW characteristic as one of our predictors, the collinearity diagnostic was conducted with respect to this characteristic. The characteristics that had severe collinearity toward HW were not used in the DFA (collinearity tolerance < 0.1; variance inflation factor (VIF) > 10) (Tables S4 and S5). As a result, only PsMxW and HW were used in further DFA together with other characteristics that had correlation coefficients less than 0.90.

**Figure 5:**
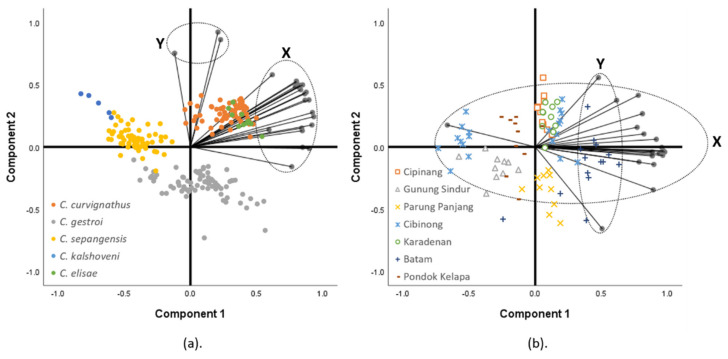
The principal component analysis biplot graph of: (**a**) all species (n = 228, 24 variables); (**b**) the group of *C. gestroi* (n = 84, 20 variables). Species are represented as different shapes. Correlated variables, which headed to the same axis direction, were projected to the collinearity diagnostics. The X variables’ clade affected the data distribution along the horizontal axis, and the Y variables clade affected the data distribution along the vertical axis.

The PCA in the group of all species found two components with sets of supportive characteristics (Figure 5). While most of the HC, trunk, and appendage characteristics loaded Component 1, the data were sufficiently explained by the seta numbers characteristics loading value in Component 2 (loading > 0.5). Both components covered 82.43% of the cumulative variance (Table S2). In the group of *C. gestroi* PCA, the distribution of data was mostly explained by Component 1, which included all sets of supportive characteristics (loading > 0.5). Component 2 did not sufficiently support the data explanation. Both components covered a cumulative variance of 76.79% (Figure 5, Table S6).

In the DFA of all species, 99.6% of grouped cases were correctly classified (cross-validated). There was a 1.2% chance that *C. gestroi* was mistakenly classified into *C. sepangensis* (Table S7), while the *C. kalshoveni* group was treated as ungrouped cases. The HC morphometrics, seta numbers, and labium characteristics were explained as important discriminative variables (loading > 0.5 in Canonical 1 and 2, Table S8). In the discrimination of *C. gestroi*, 6 of 7 grouped cases overlapped (Table S9) and there was no single characteristic that had unique discriminative ability for the data (Table S10).

### 3.3. Geometric Morphometrics

Geometric morphometric analysis was conducted to determine the magnitudes of PS and HC shape regardless of the size difference. Regression analysis was performed to remove the effects of allometry and size differences within the samples (PS, predicted: 1.5%, *p*-Value: 0.015; HC, predicted: 7.84%, *p*-Value: <0.0001) [68,81]. Shape comparison analyses (CVA and DFA) were then conducted using the residual component from the previous regression.

**Figure 6:**
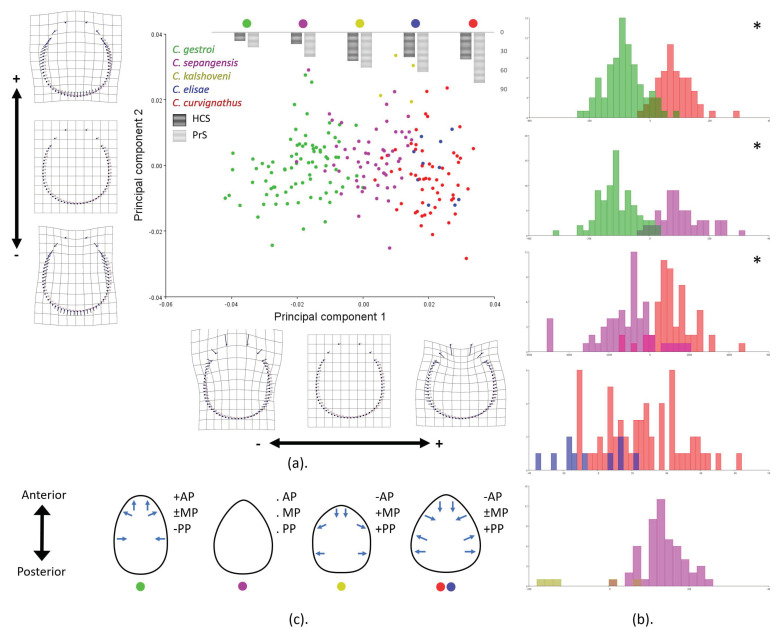
Head capsule landmarks in the group of all species were subjected to geometric morphometrics analysis using the MorphoJ program: (**a**) principal component analysis; (**b**) cross-validation score; (**c**) illustration of average pattern of head capsule shape. Species were represented in different colors. HCS: Head capsule setae, PrS: Pronotum setae. Significant *p*-Values are represented by asterisks.

In the analysis of the HC shape, 52 principal components (PCs) were extracted, and the first and second PCs together were responsible for 74.79% of the cumulative variance within the samples (PC1 = 57.24% and PC2 = 17.55%) (Table S11). It was obvious that PC1 explained the shrunken frons part (landmarks 1–9 and 52–54) and the lateral expansion of the posterior part (landmarks 11–50). A gradual lateral expansion was shown from negative to positive values across the group of all species. PC2 showed the lateral broadening of the middle part of the HC (landmarks 8–18 and 43–54) and the constriction of the most posterior margin (landmarks 22–38) (Figure 6a). Pair-wise cross-validation showed subtle differences in *C. sepangensis* and *C. curvignathus* (12.5% and 9.6% overlap, respectively), while HC shape differences in *C. elisae* and *C. curvignathus* were not obvious. Around 40% of *C. elisae* specimens fell into the *C. curvignathus* group and 14% of *C. curvignathus* specimens fell into the *C. elisae* group (Figure 6b; Table S12). Four trends of HC types were observed in the group of all species (Figure 6c).

**Figure 7:**
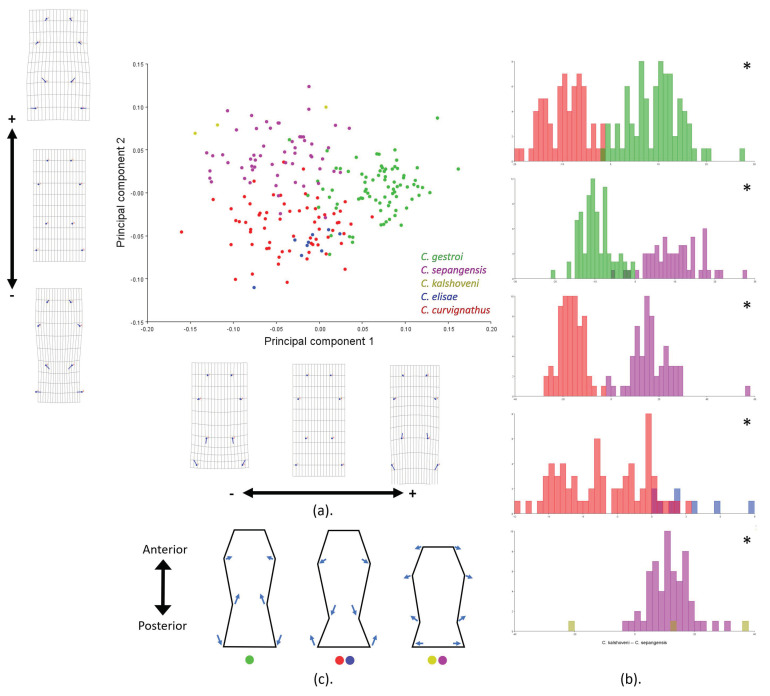
Postmentum landmarks of the group of all species were subjected to geometric morphometric analysis using the MorphoJ program: (**a**) Principal component analysis; (**b**) cross-validation score; (**c**) illustration of average pattern of the postmentum shape. Species are represented in different colors. Significant *p*-Values are represented by asterisks.

In the PS shape analysis, the first and second PCs together were responsible for 85.60% of the cumulative variance within the samples (PC1: 58.98% and PC2: 26.62%). PC1 explained the change in the PS waist point by moving away from or closer to the posterior margin (landmarks 5–8), while PC2 explained the shape lateral expansion at all landmarks (Figure 7a). CVA and discriminant analysis maximized the group ordination by using species as the criterion. Both the first two canonical variances (CV) were responsible for 93.86% of the cumulative variance (CV1: 62.67% and CV2: 31.18%) (Table S13). Cross-validation of the pairwise group showed that 12% of cases were overlapping, as *C. curvignathus* fell into the *C. elisae* PS shape group (Figure 7b; Table S14). Three trends of PS shape were observed in the analysis of all species (Figure 7c).

**Figure 8:**
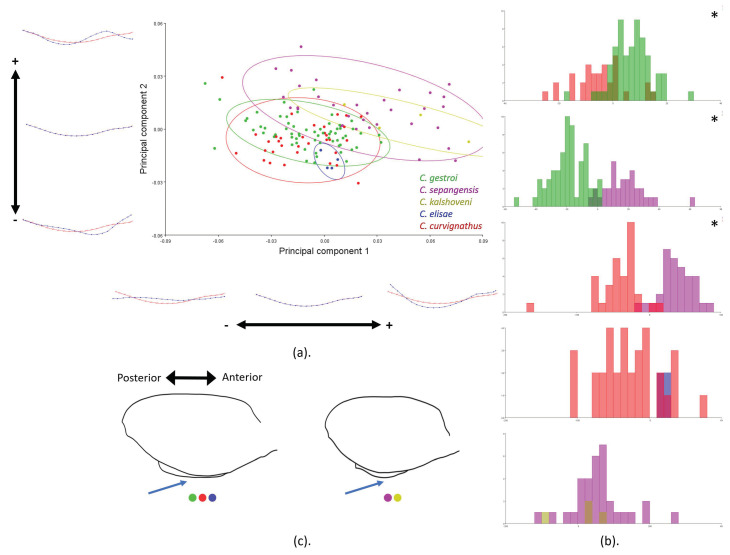
Postmentum lateral view landmarks of the group of all species were subjected to geometric morphometric analysis using the MorphoJ program: (**a**) Principal component analysis; (**b**) cross-validation score; (**c**) illustration of PS shape lateral view average pattern. Species are represented in different colors. Significant *p*-Values are represented by asterisks.

In a lateral view comparison of the PS shape in the group of all species, obvious landmark movement was demonstrated. CVA using PC1 and PC2 (86.17%) showed the landmarks in the *C. sepangensis* and *C. kalshoveni* groups and explained the ventrally bulged PS (landmarks 7–14), while the PS was relatively flattened in *C. curvignathus* and *C. gestroi* (Figure 8a,c and Table S15). The cross-validation result showed that 27% of *C. curvignathus* specimens fell into the *C. gestroi* group, and 12.7% of *C. gestroi* specimens fell into the *C. curvignathus* group. As many as 12% of *C. sepangensis* specimens fell into the *C. kalshoveni* group, and 25% of *C. kalshoveni* specimens fell into the *C. sepangensis* group. In a similar finding, a considerable number (21.2%) of *C. curvignathus* specimens were grouped into the *C. elisae* group (Figure 8b; Table S16). Two shape trends were observed in the analysis of all species. A small sample number of *C. kalshoveni* and *C. elisae* might be inadequate to be used in the significancy test. Hence, it could affect the validation result against the other species.

**Figure 9:**
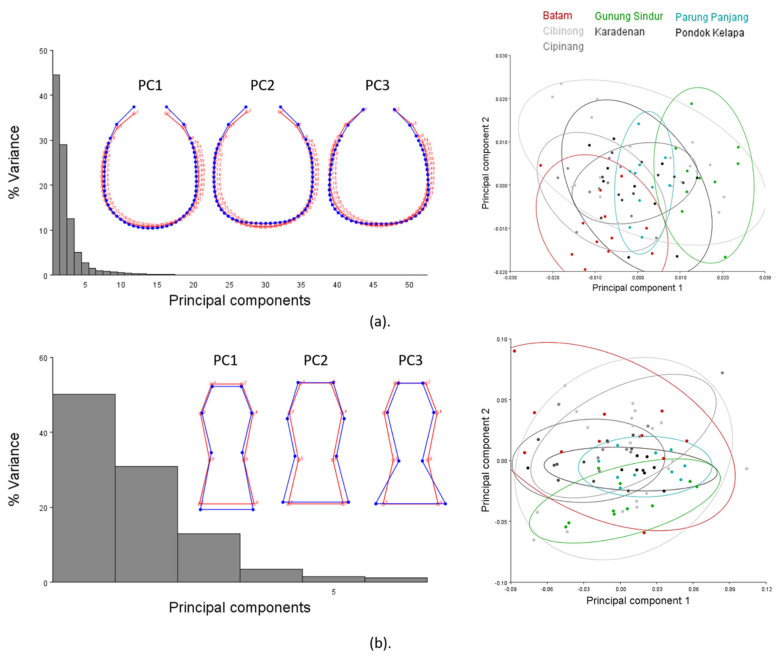
Head capsule and postmentum landmarks in the group of *C. gestroi* were subjected to geometric morphometric analysis using the MorphoJ program: (**a**) Principal component analysis of head capsule shape; (**b**) principal component analysis of postmentum shape. Functional groups based on different collection sites are represented in different colors.

Interestingly, the results of the analysis of the HC shape in the group of *C. gestroi* were consistent with the results for the PS shape analysis. Both PC1 in the two analyses respectively explained the shrinking of the middle and posterior head part (44.49%) in the inner direction (landmarks 8–20 and 40–53) and the posterior margin of the inferior part of the PS (50.09%) to the minimum waist point (landmarks 5–8). PC2 showed lateral expansion in both the head (28.98%) and PS (30.90%). The constriction of the anterior parts of the HC and PS shape was explained by each PC3 (12.57% and 12.90% respectively) (Figure 9). Some specimens collected at several locations showed extreme shapes, but they overlapped across locations. Three trends in the forms of the HC and PS shape were observed in the group of *C. gestroi*.

### 3.4. Pairwise Distance of 12 s and 16 s Genes

The amplification successfully retrieved ~440 bp of 12S sequences and ~680 bp of 16 s sequences (Tables S17 and S18). Several *Coptotermes* spp. sequences from GenBank were included. We analyzed eight populations of *Coptotermes* spp. from this study and *Odontotermes* sp. as an outgroup. In 16S pairwise distance analysis, 682 characteristics were observed. The genetic distance ranged up to 17.3%. The differences between *C. gestroi* sequences, including *C. heimi* as its synonym, ranged from 0–1.4%. The differences between different species among *Coptotermes* spp. ranged from 0.5–5.2%. Additionally, in 12S pairwise distance analysis, 442 characteristics were observed. The genetic distance ranged from 0–15%. About 0–2.0% genetic differences were calculated among *C. gestroi* populations, including *C. heimi* populations. Different species among the *Coptotermes* spp. ranged from 0.2–4.0%. The variability range from *C. elisae* to *C. curvignathus* was 0.2–0.5% in both genes. The ranges in *C. gestroi* and *C. heimi* were 1–2% in 12 s and 0.7–1.4% in 16 s. The distances from *C. kalshoveni* to *C. sepangensis* were 1.2–1.5% and 2.2–2.9% in 12 s and 16 s, respectively.

### 3.5. Phylogenetic Relationship Inferred from Mitochondrial Genes

The trees for each and combined genes were constructed with *Odontotermes* sp. as an outgroup from Termitidae. *Reticulitermes mandibularis*, *Prorhinotermes canalifrons*, *Heterotermes validus*, and *Heterotermes malabaricus* were used as outgroups as members of Rhinotermitidae. *Coptotermes heimi*, which was suggested to be a junior synonym of *C. gestroi* [40], was also included in the analysis. The tree topology resulting from combined genes analysis produced seven clades of *Coptotermes* spp. with strong probability.

**Figure 10:**
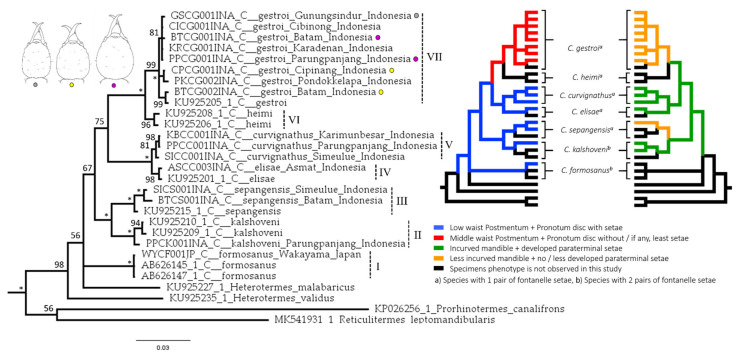
The tree inferred from combined 16 s and 12 s rRNA gene fragments (~1127 bp) using MrBayes 3.2.7. The sequences with sample codes were taken from the NCBI database. A 100% probability is represented by an asterisk (*) at the node. The Roman numerals represent clades, and the three extreme shape differences in the group of *C. gestroi* are shown in the different gray, purple, and yellow circles in the phylogeny tree. The pair of characteristics is displayed using different colors, while the pairing of fontanelle setae characteristics is shown using the superscripts “a” and “b”.

Clade I included *C. formosanus* from Iriomote Island and the Okinawa Islands nested with the sample from Wakayama, Japan [82]. Clade II consisted of *C. kalshoveni* from Singapore and Thailand together with a sample of *C. kalshoveni* from Parung Panjang, Indonesia. Clade III consisted of *C. sepangensis* from Brunei [83] and *C. sepangensis* from Simeulue Island and Batam Islands, Indonesia. Clade IV included *C. curvignathus* from Karimunbesar Island, Parung Panjang, and Simeulue Island, Indonesia, and Clade V contained *C. elisae* from Asmat regency, Indonesia, and New Guinea [83]. Clade VI included *C. Heimi* from Pakistan [83], and Clade VII contained all *C. gestroi* populations from this study nested together with *C. gestroi* from Singapore [83]. The combined genes tree provided by a Bayesian inference was similar to the maximum likelihood tree topology using 12S and 16S fragments (Figures S1 and S2). The only difference was that *C. formosanus* was grouped with *H. validus* and was the closest group to the rest of the *Coptotermes* spp. in the maximum likelihood test of 16S. The tree in this study is similar to those in other studies in which *Heterotermes* nestled among *Coptotermes* [48,83,84] (Figure 10).

**Figure 11:**
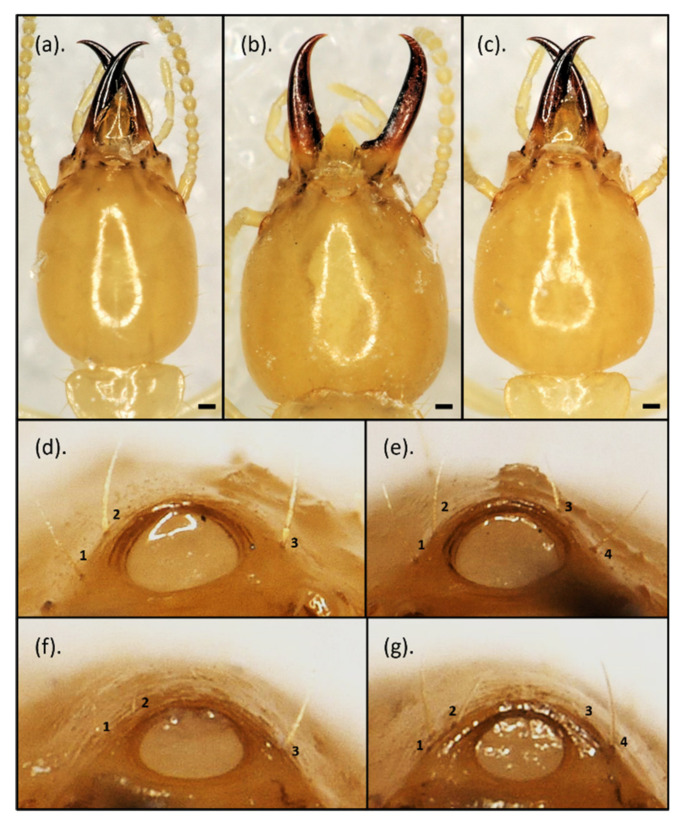
The three extreme head capsule shapes of *Coptotermes gestroi* were explained by geometric morphometric analysis and by variation in the number of fontanelle setae. (**a**–**c**): Specimens from (**a**) Gunung Sindur, (**b**) Batam Island, and (**c**) Parung Panjang populations: (**d**) *Coptotermes curvignathus* with three fontanelle setae, (**e**) *C. curvignathus* with four fontanelle setae, (**f**) *C. gestroi* with three fontanelle setae, and (**g**) *C. gestroi* with four fontanelle setae. The pictures were taken on the same scale.

The tree may also explain how the group of characteristics was formed (Figure 10). *Coptotermes gestroi* evolved as a monophyletic clade in *Coptotermes* spp. sharing the characteristics of a middle-waist PS and less-developed setae on the pronotum disc. The other *Coptotermes* in this study possessed a low-waist PS accompanied by a pronotum with setae on its disc. A less-incurved mandible and less-developed para-terminal setae were independently evolved. Although *C. kalshoveni* was represented by a small number of specimens, it was obvious that each *C. kalshoveni* specimen had two pairs of setae around the fontanelle and could be discriminated from other *Coptotermes* observed in this study. There was, however, a small chance that *C. gestroi* and *C. curvignathus* specimens exhibiting two pairs of fontanelle setae were found in nature (Figure 11).

## 4. Discussion

### 4.1. Important Noticeable Characteristics

*Coptotermes* (Wasmann) is an exceptional group of Rhinotermitidae that is decorated by well-developed dome-like frons ending in a large opening fontanelle [85,86]. The number of setae around the fontanelle is a very important characteristic that discriminates *C. formosanus* from *C. gestroi* in regions with overlapping swarms, such as Florida (U.S.) [87,88,89] and Taiwan [90]. *C. gestroi* has been known to have a pair of setae around the fontanelle, while *C. formosanus* has two pairs of setae [87,88]. Although we observed a few variations in *C. gestroi* (2–4 setae), *C. sepangensis* (2–3 setae), and *C. curvignathus* (1–4 setae) (Figure 11), most of the specimens observed had one pair of fontanelle setae. This variation of fontanelle setae was also observed on the soldiers of laboratory cultured incipient colony [91]. *Coptotermes kalshoveni* exhibited two pairs of fontanelle setae across five observed specimens.

The seta arrangement on the upper face of the labrum varies among *Coptotermes* spp. [92]. It also has been well-illustrated in several identification references [26,52,53,93]. In this study, variation in lengths of the para-terminal setae discriminated two groups of species, with *C. gestroi* and *C. kalshoveni* having one pair that is relatively less-developed compared to those of *C. elisae*, *C. curvignathus,* and *C. sepangensis* (Figure 3). The less-developed para-terminal setae coexisted with a less incurved mandible (Figure 10).

Apart from showing their characteristics’ importance, multicollinearity of size characteristics concealed other variables that were also important, such as the PrS and head-capsule setae (HCS). We found PrS was an important characteristic for the species discrimination process in this study. Considering the pronotum pilosity and PS waist in *C. formosanus* as described by Chen (2020) [94], the absence of setae on the pronotum disc together with the middle PS waist characteristic defined a monophyletic group that may discriminate *C. gestroi* from the other species observed in this study (Figure 3 and Figure 10). The characteristics of setae on the abdomen, labrum and pronotum were also used to discriminate the *Odontetermes*, *Heterotermes* and *Reticulitermes* species complex [28,29,30,31,32].

The size characteristics have been used as primary key species identifiers within the *Coptotermes* genus [23,24,26,51,53]. However, as shown in this study, depending solely on the massive HC size as a characteristic of *C. curvignathus* has led to confusion with that of *C. gestroi*. The *C. gestroi* from Batam Island was observed to have the largest HC among *C. gestroi* in this study. The range of HLBM sizes was close to that of Roonwal and Chhotani’s collection from Assam, while the HW was close to that of Kalshoven’s collection from Java [24,95]. The *C. gestroi* of this size was observed to overlap with that of *C. curvignathus*, and the typical overlap measurements have also been observed in other studies [22,23,27,52,57,96]. Mandible curvature consequently has been suggested as the diagnostic characteristic with which to discriminate them [22,57].

Furthermore, the PS of *Coptotermes* spp. has been well-described in detailed drawings showing the waist appearance in the studied species [24,26,53]. Roonwal and Chhotani [26] measured two head height parameters by either including or excluding the PS to indicate its emergence from the HC. This study also observed a difference in the PS shape in the lateral view of *C. gestroi, C. curvignathus,* and *C. elisae* against *C. sepangensis* and *C. kalshoveni*. The latter pair demonstrated a ventrally bulged surface compared to the former pair (Figure 7). Our current study suggested that PS shape (Figure 7) in the ventral view is an additional important characteristic for discriminating *C. elisae* and *C. curvignathus* from *C. gestroi*, while PS shape in the lateral view (Figure 8) can be used to discriminate *C. elisae*, *C. curvignathus,* and *C. gestroi* from *C. sepangensis* and *C. kalshoveni*. Thapa’s *C. kalshoveni* descriptions and Tho’s suggestion to use mandible curvature to distinguish them from *C. sepangensis* were also clearly feasible [27,52]. It was confirmed in the present study that *C. kalshoveni* has a less incurved mandible compared to that of *C. sepangensis* (Figure 3).

### 4.2. Putative Function-Related Shape and Size Variation of HC and PS

The geometric morphometric analysis demonstrated the interspecific variation of the HC shape within the group of all species (Figure 6a). In another study, HW of *Reticulitermes speratus* was demonstrated to be important in the phragmotic defense by showing a smaller coefficient of variation (CV) compared to the other body parts [97]. Among all species observed in this study, only HW of *C. curvignathus, C elisae* and *C. sepangensis* showed smaller CV compared to other head-part measurements (Wikantyoso, unpublished data).

The experiment conducted by Li et al. [89] demonstrated *C. gestroi* to be more attacking and aggressive in interspecific competition compared to *C. formosanus.* In this study, setae numbers in *Coptotermes* spp. increased as the posterior HC laterally expanded. Hairlike setae or sensilla with specific cuticular profile may sense vibration or tactical stimulation [98,99,100,101,102,103]. Hence, species with more setae might evolve to have better ability to sense vibration from their surroundings, such as performing an eavesdropping mechanism to detect the nearest competitor or predator-walking impulse [104,105]. Based on this information, the *Coptotermes* soldier might evolve so that the species with more setae and laterally expanded HC would be more sensitive to vibro–acoustic cues and have greater advantage of phragmosis in the tunnel defense, while the one with fewer setae and slender HC would be more impulsive and rush their natural competitor or predator during interception.

The HC shape and mandible are suggested to become an intact set of characteristics in the evolution process [106]. Different levels of mandible curvature independently evolved in *Coptotermes* and were not correlated with the HC posterior lateral expansion. Lateral expansion of the HC posterior part may be associated with the wider attachment area of the craniomandibular muscle that will amplify bite force [106,107,108]. We assumed craniomandibular muscle development affects the postmentum elongation and HC widening during soldier differentiation in *Coptotermes*. The structure of the mandible closer-muscle in *Hodotermopsis sjostedti* (*bn4*) seems to have a role in constraining the postmentum waist [20], of which praementum retraction muscles attach [109,110].

### 4.3. Impact of Coptotermes Head Shape Perplexity on Indonesia Termite Pest Management

Termite pest determination strongly depends on the existence of a white wax-like secretion of the large frontal hole, which is usually used to discriminate *Coptotermes* from other genera. However, the assessment of head-shape characteristics is crucial when it comes to species description [86,94]. Not only is the sclerotized part able to withstand preservation, but HC of termites were unpalatable to their predator of equal size [18,111].

In Hanoi, Vietnam, termite pests were identified with two types of HC shapes that genetically fall in the same phylogenetic clade of *C. gestroi* [21]. In the present study, three extreme types of head shapes that constitute more than 90% of the variance in data samples were nested together. A population from Gunung Sindur showed a shrinking middle-head part that resembled an oval or egg-shaped HC, which is similar to that of *C. gestroi* from Hanoi (C2 in Hanh et al. [21]). In the other specimens from Parung Panjang and Batam Island, the posterior part of the head was laterally broadened to resemble the shape of a waterdrop. Despite the subjectivity in determining these shapes, head-shape variation is widely considered to be a striking characteristic of the HC in *C. gestroi* and *C. curvignathus* during field assessment [112,113,114,115,116]. Hence, relying solely on HC shape is not sufficient for termite pest determination on the field.

Notably in this study, *C. elisae* and *C. curvignathus* had overlapping HC and PS shapes in both the ventral and lateral view analyses. Genetic analysis also explained small genetic distance (below 0.5 in both 12 s and 16 s) between the two species. Furthermore, *C. heimi* and *C. vastator* were suggested to be *C. gestroi* synonyms due to their overlapping morphometric characteristics and low genetic variation (below 1.7% and 0.54% in 12 s, respectively; 1.4% and 0.8% in 16 s, respectively) [40,49]. Based on the evidence, we believe that *C. elisae* and *C. curvignathus* may be synonyms, even though additional thorough evaluations are needed to validate this possibility. This was also proposed by Chouvenc et al. [10], who marked *C. curvignathus* as nonvalid species and suspected the synonymy of *C. elisae* and *C. curvignathus* from their morphology and genetic identity in Bourguignon’s unpublished data. The synonym issue of both species could have been affecting the portrait of termite pest distribution in Indonesia.

The distribution of economically important termite pests in the Indonesian archipelago was still globally uncertain [10], while the local mapping of economically important species in several important urban areas in Indonesia was recently conducted [113,116,117]. Likewise, the morphometrics information was still poorly explained. Our data suggested that the shape variation showed a subtle interspecific shape trend in the group of all species, while the HC shape variation in *C. gestroi* might not be geographically specific. Instability characteristics in *C. gestroi* soldiers may explain the more environmental stress they get compared to other species in this study [91], such as urban hustle. On the other hand, *C. gestroi* soldiers evolved morphological traits distinct from others that might partially ease their colony expansion in urban areas, such as less hairlike setae on the pronotum and HC. The small variance value (Wikantyoso, unpublished data) and lopsided distribution of pronotum disc setae characteristics should potentially illustrate the directional selection on the *C. gestroi* soldier caste (Figure 4). Some insects have had their distribution, behavior, and reproduction affected by anthropogenic activities [118,119,120,121]. The coexistence of the broadening of the posterior part of the head with the increasing of the HC and PrS or hairlike sensilla might explain the predator-based defense mechanism and the sensitivity to natural predator or human-induced noise, due to which each species could express a distribution preference within urban, suburban, and forest areas in Indonesia.

## 5. Conclusions

This study did not aim to revise the classification of *Coptotermes* but rather to explore the complexity of the determination of *Coptotermes* spp. pest species using head-capsule (HC) characteristics. Head width (HW), pronotum setae (PrS), and postmentum (PS) characteristics appeared to be important in the discrimination process. Four trends of HC shape were observed with lateral expansion of HC posterior part happening in a gradual manner in *C. gestroi, C. sepangensis,* and *C. curvignathus*, successively in that order. Three possible extreme variations were shown to exist in *C. gestroi* populations in Indonesia, and these variations explained the confusion in *C. gestroi* species determination. Considering both the ease with which the HC shape can be observed during field observations and the grouping results in this study, HC shape should be cautiously used to discriminate among *Coptotermes* spp. In the absence of a species comparison in the field, it is suggested that PrS and PS shape be used rather than linear measurements.

## Figures and Tables

**Table 1:** Specimens list from the study and several sequences from other studies that were included in the genetic analysis. * CMG: Complete mitochondrial genome.

Sample Code	Species	Collecting Site	GenBank Accession Number		
			12S	16S	CMG *
	Samples from This Study				
CPCG001INA	*C. gestroi*	Cipinang, Indonesia	MW765243	MW765223	
GSCG001INA	*C. gestroi*	Gunung Sindur, Indonesia	MW765244	MW765224	
KRCG001INA	*C. gestroi*	Karadenan, Indonesia	MW765246	MW765226	
PPCG001INA	*C. gestroi*	Parung Panjang, Indonesia	MW765247	MW765227	
CICG001INA	*C. gestroi*	Cibinong, Indonesia	MW765248	MW765228	
PKCG002INA	*C. gestroi*	Pondok Kelapa, Indonesia	MW765250	MW765230	
BTCG001INA	*C. gestroi*	Batam, Indonesia Col. 1	MW765245	MW765225	
BTCG002INA	*C. gestroi*	Batam, Indonesia Col. 2	MW765249	MW765229	
BTCS001INA	*C. sepangensis*	Batam, Indonesia	MW765251	MW765231	
SICS001INA	*C. sepangensis*	Alafan Simeulue, Indonesia	MW765252	MW765232	
PPCK001INA	*C. kalshoveni*	Parung Panjang, Indonesia	MW765254	MW765234	
PPCC001INA	*C. curvignathus*	Parung Panjang, Indonesia	MW765255	MW765235	
SICC001INA	*C. curvignathus*	East Simeulue, Indonesia	MW765256	MW765236	
KBCC001INA	*C. curvignathus*	Karimunbesar, Indonesia	MW765257	MW765237	
ASCC003INA	*C. elisae*	Asmat, Papua, Indonesia	MW765260	MW765240	
WYCF001JP	*C. formosanus*	Wakayama, Japan	MW765261	MW765241	
CIOJ001INA	*Odontotermes* sp.	Cibinong, Indonesia	MW765262	MW765242	
	**Samples from other studies**				
	*C. gestroi*	Singapore			KU925205
	*C. heimi*	Pakistan			KU925206
	*C. heimi*	Pakistan			KU925208
	*C. sepangensis*	Brunei			KU925215
	*C. kalshoveni*	Singapore			KU925209
	*C. kalshoveni*	Thailand			KU925210
	*C. formosanus*	Okinawa Island, Okinawa			AB626145
	*C. formosanus*	Iriomote Island, Okinawa			AB626147
	*H. malabaricus*	India			KU925227
	*H. validus*	Australia			KU925235
	*P. canalifrons*	Réunion Island, France			KP026256
	*R. leptomandibularis*	Zhejiang, China			MK41931

## Data Availability

The data presented in this study are available on request from the corresponding author.

## Supplementary Materials

The following are available online at https://www.mdpi.com/article/10.3390/insects12050477/s1, Figure S1: Tree inferred from 12S (A) and 16S (B) rRNA gene fragment by using MeBayes 3.2.7. N-generation was 10 × 10^6^ with 3 × 10^5^ burn-in. Substitution model was GTR_G for each gene. Sequences with simple code were taken from NCBI database. A hundred percent probability was represented by asterice (*) at the node, Figure S2: Maximum Likelihood Tree inferred from 12S (A) and 16S (B) rRNA gene fragment by using Mega 5.2. Substitution model was GTR_G for each gene and 1000 × bootstrap replication. Sequences with sample code were taken from NCBI database, Table S1: Measurements of 5 morphospecies observed in this study. *numbers (continued), Table S2: Rotated Component Matrix by Varimax. Two components and seven sets of character were explained. Total % of Variance covered 82.43%, Table S3: Characteristics comparison of Coptotermes spp, Table S4: Collinearity diagnostics conducted by Linear Regression analysis. Tolerance value < 0.1 or VIF > 10.00 explained collinearity. Head width character was treated as dependent variable, Table S5: Collinearity diagnostics conducted by Linear Regression analysis. Tolerance value < 0.1 or VIF > 10.00 explained collinearity. Head width character was treated as dependent variable, Table S6: Rotated Component Matrix by Varimax. Two components were extracted. Five sets of character were explained. Total % of Variance covered were 76.79%, Table S7: The classification results obtained by discriminant function analysis for all of the species. Original and cross-validated classifications were included. Overall, 99.5% of groupings were solved by 11 variables. Cc: *C. curvignathus*; Cg: *C. gestroi*; Cs: *C. sepangensis*; Ce: *C. elisae*, Table S8: Standardized canonical discriminant matrix of whole species DFA. Two components were extracted. Five sets of character were explained. Total % of Variance covered by two components was 100%, Table S9: titleThe classification results obtained by discriminant function analysis for C. gestroi species with head morphology variation. Original and cross-validated classifications were included. Overall, 82.1% of groupings were step-wisely solved by engaging nine variables. CP: Cipinang; GS: Gunung Sindur; PP: Parung Panjang; CI: Cibinong; KR: Karadenan; BT: Batam; PK: Pondok Kelapa, Table S10: Standardized canonical discriminant matrix by DFA for C. gestroi with head morphology variation. Six components were extracted. Six sets of character were explained. Total % of Variance covered by the first two components was 64%, Table S11: The amount of data variance covered by the four highest Canonical Variate produced from CVA of the whole species Head Capsule Shape. The number of iterations is 10,000, Table S12: Head Capsule Shape Classification results by DFA for the whole species. In Head Capsule shape analysis, comparisons were done in pairwise way (CC: *C. curvignathus*; CE: *C. elisae*; CG: *C. gestroi*; CK: *C. kashoveni*; CS: *C. sepangensis*), Table S13: The amount of data variance covered by three Canonical Variate produced from CVA of the whole species Postmentum Shape. The number of iterations is 10,000, Table S14: Postmentum Shape Classification results by DFA for the whole species. In Postmentum shape analysis, comparisons were done in pairwise way (CC: *C. curvignathus*; CE: *C. elisae*; CG: *C. gestroi*; CK: *C. kashoveni*; CS: *C. sepangensis*), Table S15: The amount of data variance covered by the four highest Canonical Variate produced from CVA of the whole species Posmentum Lateral View. The number of iterations is 10,000, Table S16: Postmentum lateral view classification results by DFA for the whole species. In Head Capsule shape analysis, comparisons were done in pairwise way (CC: *C. curvignathus*; CE: *C. elisae*; CG: *C. gestroi*; CK: *C. kashoveni*; CS: *C. sepangensis*), Table S17: Pairwise distance matrix (uncorrected p-distances %) for all the 12S sequence used in this study, Table S18. Pairwise distance matrix (uncorrected p-distances %) for all the 16S sequence used in this study.
