# Supplementary material for: Morphometric Analysis of Coptotermes spp. Soldier Caste (Blattodea: Rhinotermitidae) in Indonesia and Evidence of Coptotermes gestroi Extreme Head-Capsule Shapes
Source: Insects. 2021 May 20;12(5):477. doi: 10.3390/insects12050477 (PMC8160883; doi:10.3390/insects12050477)
Supplement: Supplementary file 1 [file insects-12-00477-s001.zip › Figure S1.pdf]

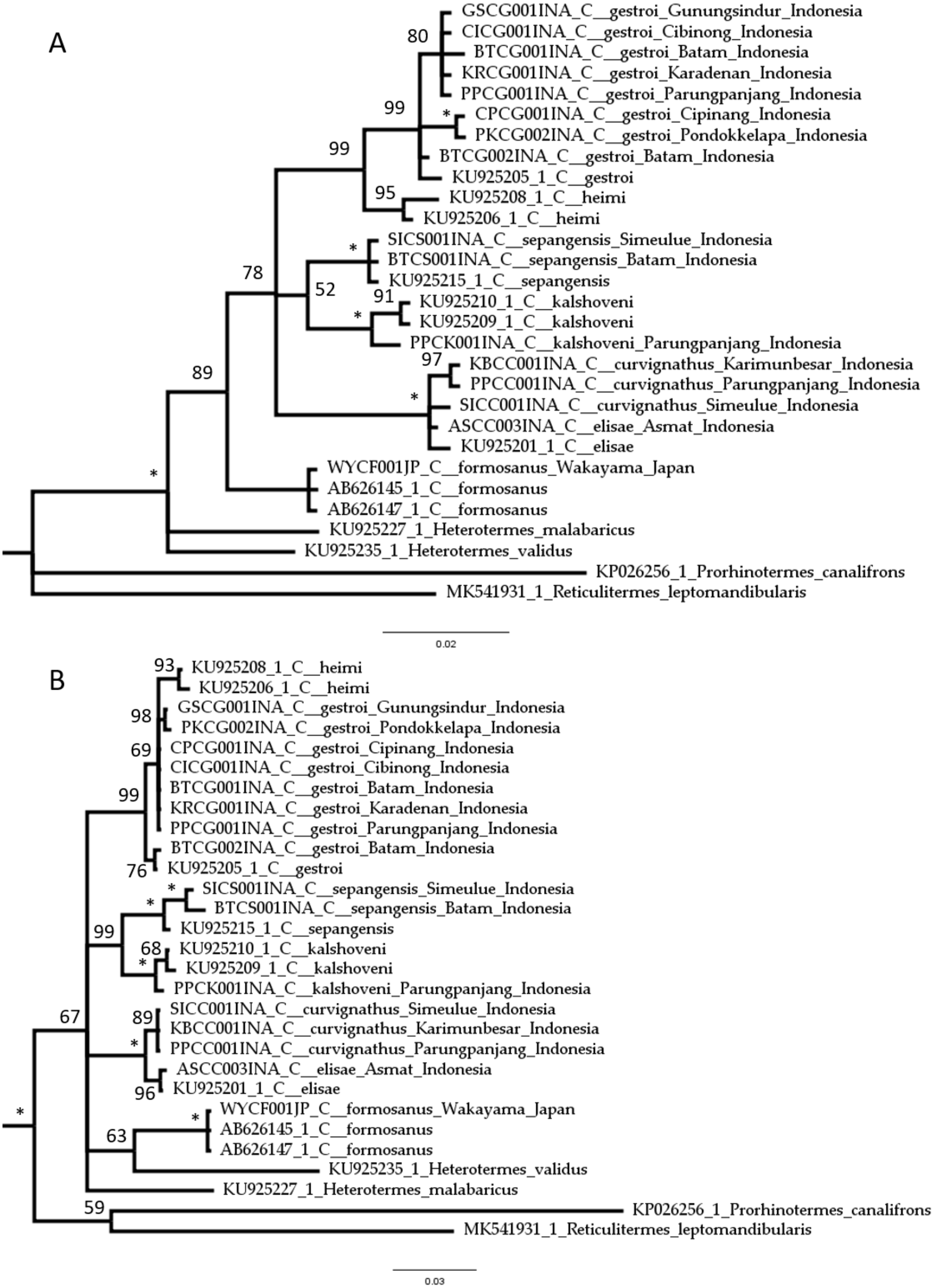

Figure S1. Tree inferred from 12S (A) and 16S (B) rRNA gene fragment by using MrBayes 3.2.7. N-generation was 10 x 10^6 with 3 x 10^5 burn-in. Substitution model was GTR_G for each gene. Sequences with sample code were taken from NCBI database. A hundred percent probability was represented by asterice (*) at the node.
