# Supplementary material for: Morphometric Analysis of Coptotermes spp. Soldier Caste (Blattodea: Rhinotermitidae) in Indonesia and Evidence of Coptotermes gestroi Extreme Head-Capsule Shapes
Source: Insects. 2021 May 20;12(5):477. doi: 10.3390/insects12050477 (PMC8160883; doi:10.3390/insects12050477)
Supplement: Supplementary file 1 [file insects-12-00477-s001.zip › Figure S2.pdf]

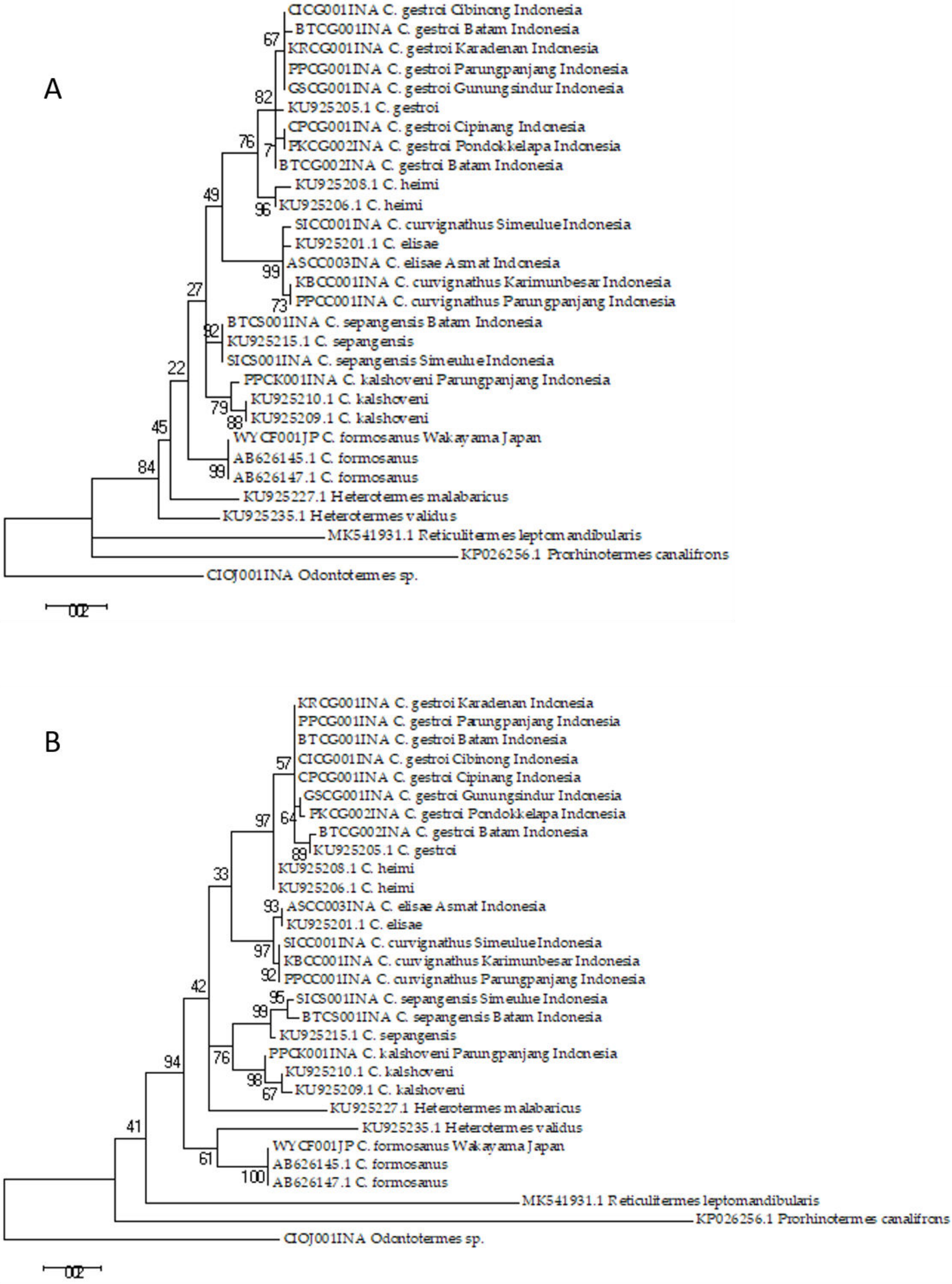

Figure S2. Maximum Likelihood Tree inferred from 12S (A) and 16S (B) rRNA gene fragment by using Mega 5.2. Substitution model was GTR_G for each gene and 1000 x bootstrap replication. Sequences with sample code were taken from NCBI database.
