# Supplementary material for: Morphometric Analysis of Coptotermes spp. Soldier Caste (Blattodea: Rhinotermitidae) in Indonesia and Evidence of Coptotermes gestroi Extreme Head-Capsule Shapes
Source: Insects. 2021 May 20;12(5):477. doi: 10.3390/insects12050477 (PMC8160883; doi:10.3390/insects12050477)
Supplement: Supplementary file 1 [file insects-12-00477-s001.zip › Table S1.pdf]

Table S1. Measurements of 5 morphospecies observed in this study. (*numbers, ***not subjected into multivariate analysis)

	Characters (μm)	*C. curvignathus* (n = 66; **112)				*C. gestroi* (n = 84; **152)				*C. sepangensis* (n = 63; **126)				*C. kalshoveni* (n = 5; **8)			
		**Mean**	**Min.**		**Max.**	**Mean**	**Min.**		**Max.**	**Mean**	**Min.**		**Max.**	**Mean**	**Min.**		**Max.**
1	Head length to the tip mandible (HLM)	2396.83 ± 19.24	2031.00	-	2677.00	2017.36 ± 15.66	1754.00	-	2322.00	1726.05 ± 13.98	1314.00	-	1928.00	1635.20 ± 31.50	1564.00	-	1733.00
2	Head length to the base mandible (HLBM)	1568.62 ± 10.66	1380.00	-	1745.00	1303.67 ± 10.99	1100.00	-	1510.00	1165.27 ± 6.99	997.00	-	1320.00	1107.00 ± 12.16	1066.00	-	1127.00
3	Base mandible to the tip mandible length (BML)	827.09 ± 13/78	550.00	-	1069.00	709.33 ± 7.36	564.00	-	892.00	567.70 ± 7.38	422.00	-	705.00	526.20 ± 27.22	460.00	-	606.00
4	Head width (HW)	1405.97 ± 9.20	1205.00	-	1531.00	1093.17 ± 11.02	890.00	-	1322.00	1009.71 ± 5.74	830.00	-	1143.00	976.40 ± 5.48	964.00	-	992.00
5	Head width on the base mandible (HWBM)	789.11 ± 4.60	711.00	-	905.00	659.36 ± 5.04	559.00	-	763.00	545.90 ± 3.12	465.00	-	614.00	468.80 ± 8.13	450.00	-	492.00
6	Head capsule seta (HCS)*	42.48 ± 0.79	31.00	-	56.00	13.71 ± 0.39	2.00	-	23.00	18.52 ± 0.51	13.00	-	32.00	45.20 ± 3.84	31.00	-	53.00
7	Fontanel width (FW)	235.52 ± 3.07	187.00	-	294.00	184.71 ± 1.80	132.00	-	217.00	184.41 ± 1.81	154.00	-	217.00	180.60 ± 1.94	174.00	-	186.00
8	Labrum maximum width (LbMxW)	315.92 ± 2.58	228.00	-	349.00	277.81 ± 3.27	202.00	-	333.00	268.44 ± 33.37	185.00	-	2331.00	221.20 ± 3.67	213.00	-	231.00
9	Labrum maximum length (LbMxL)	404.55 ± 6.18	279.00	-	495.00	362.13 ± 4.64	279.00	-	459.00	259.71 ± 4.82	157.00	-	330.00	224.40 ± 9.34	193.00	-	248.00
10	Labrum Paraterminal Setae Length (LbPtSL)**	32.56 ± 1.58	8.90	-	130.30	14.11 ± 0.31	5.20	-	24.41	30.17 ± 1.22	9.65	-	78.80	13.43 ± 0.72	10.48	-	15.83
11	Left mandible length (LML)	1067.05 ± 5.29	962.00	-	1164.00	846.56 ± 5.96	730.00	-	976.00	744.48 ± 5.84	588.00	-	830.00	655.60 ± 5.11	645.00	-	672.00
12	Head height (HHt)	998.91 ± 7.92	869.00	-	1155.00	836.14 ± 7.76	711.00	-	982.00	792.57 ± 3.80	732.00	-	859.00	738.00 ± 7.91	719.00	-	767.00
13	Postmentum median length (PsMdL)	949.24 ± 8.67	735.00	-	1050.00	871.44 ± 6.43	743.00	-	1019.00	636.65 ± 7.12	490.00	-	732.00	590.60 ± 21.33	521.00	-	647.00
14	Postmentum maximum width (PsMxW)	421.86 ± 2.54	374.00	-	476.00	383.00 ± 3.11	318.00	-	450.00	337.37 ± 2.23	305.00	-	392.00	329.60 ± 3.37	319.00	-	339.00
15	Postmentum minimum width (PsMnW)	236.30 ± 1.73	205.00	-	264.00	248.50 ± 3.20	199.00	-	321.00	227.63 ± 1.76	185.00	-	257.00	257.00 ± 4.20	246.00	-	267.00
16	Postmentum anterior width (PsAW)	248.76 ± 2.55	196.00	-	297.00	222.76 ± 2.54	119.00	-	267.00	188.51 ± 1.79	156.00	-	225.00	193.80 ± 5.91	178.00	-	211.00
17	Postmentum seta (PsS)*	10.45 ± 0.40	4.00	-	18.00	6.32 ± 0.24	1.00	-	15.00	8.84 ± 0.27	4.00	-	15.00	12.20 ± 0.37	11.00	-	13.00
18	Pronotum median length (PrMdL)	516.17 ± 6.26	407.00	-	585.00	396.71 ± 4.68	302.00	-	496.00	355.51 ± 3.82	296.00	-	421.00	339.00 ± 4.24	325.00	-	351.00
19	Pronotum maximum length (PrMxL)	576.05 ± 6.45	458.00	-	649.00	428.62 ± 5.20	320.00	-	538.00	394.97 ± 4.64	323.00	-	473.00	387.80 ± 4.07	376.00	-	397.00
20	Pronotum maximum width (PrMxW)	992.39 ± 9.01	813.00	-	1091.00	769.99 ± 9.98	579.00	-	1001.00	699.38 ± 6.29	613.00	-	818.00	647.20 ± 9.35	613.00	-	665.00
21	Pronotum seta*(PrS)	80.09 ± 1.36	61.00	-	113.00	23.54 ± 0.47	14.00	-	36.00	38.95 ± 0.73	26.00	-	58.00	55.60 ± 3.44	46.00	-	64.00
22	Pronotum margin seta (PrMS)***	44.79 ± 0.74	34.00	-	63.00	23.11 ± 0.42	13.00	-	33.00	26.40 ± 0.52	18.00	-	36.00	39.20 ± 2.15	34.00	-	46.00
23	Pronotum disc seta (PrMS)***	35.30 ± 1.02	17.00	-	54.00	0.54 ± 0.13	0.00	-	5.00	12.43 ± 0.45	6.00	-	25.00	16.40 ± 1.96	10.00	-	21.00
24	Mesonotum maximum width (MsMxW)	874.97 ± 6.36	747.00	-	949.00	703.20 ± 7.41	559.00	-	871.00	682.86 ± 5.41	610.00	-	793.00	651.20 ± 6.64	638.00	-	676.00
25	Metanotum maximum width (MtMxW)	994.24 ± 6.85	870.00	-	1118.00	822.65 ± 9.79	651.00	-	1034.00	793.84 ± 4.87	728.00	-	880.00	764.20 ± 8.02	740.00	-	783.00

Table S1. Measurements of 5 morphospecies observed in this study. *numbers (continued)

	Characters (μm)	*C. curvignathus* (n = 76)				*C. gestroi* (n = 84)				*C. sepangensis* (n = 63)				*C. kalshoveni* (n = 5)			
		**Mean**	**Min.**		**Max.**	**Mean**	**Min.**		**Max.**	**Mean**	**Min.**		**Max.**	**Mean**	**Min.**		**Max.**
26	Hind femora length (HiFL)	1068.26 ± 7.76	873.00	-	1183.00	830.75 ± 8.95	547.00	-	1040.00	764.48 ± 6.10	541.00	-	866.00	745.60 ± 14.85	710.00	-	783.00
27	Hind tibia length (HiTL)	1187.65 ± 8.78	1037.00	-	1368.00	915.48 ± 10.83	709.00	-	1114.00	857.62 ± 5.42	784.00	-	973.00	824.80 ± 11.92	796.00	-	855.00
28	Total body length without Head (TBLH)	3159.80 ± 95.97	1803.00	-	4717.00	3016.96 ± 46.48	2141.00	-	3951.00	2102.97 ± 54.68	1294.00	-	3541.00	1578.00 ± 157.96	1188.00	-	2078.00
29	Head - mandible width difference (ΔHMW)***	501.58 ± 9.72	347.00	-	680.00	457.11 ± 8.34	307.00	-	712.00	420.79 ± 6.25	296.00	-	547.00	451.40 ± 11.47	411.00	-	479.00
30	Head length - head width (ΔHLHW)***	162.65 ± 5.55	48.00	-	310.00	210.50 ± 5.45	44.00	-	313.00	155.56 ± 4.14	73.00	-	241.00	130.60 ± 10.71	102.00	-	162.00
31	Head index (HW/HLBM)***	0.90 ± 0.00	0.82	-	0.97	0.84 ± 0.00	0.77	-	0.97	0.87 ± 0.00	0.80	-	0.93	0.88 ± 0.01	0.86	-	0.90
32	Head contraction index (HW/HWBM)***	0.56 ± 0.00	0.52	-	0.65	0.61 ± 0.00	0.52	-	0.74	0.54 ± 0.00	0.49	-	0.59	0.48 ± 0.01	0.46	-	0.50
33	Mandible index (LML/HLBM)***	0.68 ± 0.00	0.59	-	0.76	0.65 ± 0.00	0.51	-	0.73	0.64 ± 0.00	0.52	-	0.70	0.59 ± 0.01	0.57	-	0.61
34	Postmentum index (PsNW/PsMW)***	0.56 ± 0.00	0.47	-	0.62	0.65 ± 0.00	0.55	-	0.78	0.68 ± 0.00	0.58	-	0.75	0.78 ± 0.01	0.74	-	0.82
35	Pronotum index (PrMW/HW)***	0.71 ± 0.00	0.63	-	0.75	0.70 ± 0.00	0.63	-	0.81	0.69 ± 0.00	0.64	-	0.84	0.66 ± 0.01	0.64	-	0.69
36	Mesonotum index (MsMW/PrMW)***	0.92 ± 0.01	0.82	-	1.16	0.94 ± 0.01	0.83	-	1.10	0.98 ± 0.00	0.91	-	1.04	1.01 ± 0.01	0.97	-	1.04
37	Metanotum index (MtMW/PrMW)***	1.01 ± 0.01	0.92	-	1.13	1.00 ± 0.01	0.89	-	1.14	1.02 ± 0.02	0.83	-	1.23	1.18 ± 0.01	1.16	-	1.21

Table S1. Measurements of 5 morphospecies observed in this study. *numbers (continued)

	Characters (μm)	*C. elisae* (n = 10; **20)			
		**Mean**	**Min.**		**Max.**
1	Head length to the tip mandible (HLM)	2555.5 ± 51.52	2306	-	2709
2	Head length to the base mandible (HLBM)	1621.2 ± 18.60	1538	-	1742
3	Base mandible to the tip mandible length (BML)	934.4 ± 44.46	702	-	1077
4	Head width (HW)	1489.6 ± 10.53	1444	-	1555
5	Head width on the base mandible (HWBM)	844.3 ± 7.31	794	-	875
6	Head capsule seta (HCS)*	39.1 ± 1.41	33	-	48
7	Fontanel width (FW)	234.8 ± 3.22	221	-	245
8	Labrum maximum width (LbMxW)	349.2 ± 4.22	326	-	369
9	Labrum maximum length (LbMxL)	426.3 ± 5.19	393	-	444
10	Labrum Paraterminal Setae Length (LbPtSL)**	31.6465 ± 4.46	13.7	-	105.2
11	Left mandible length (LML)	1059.6 ± 19.75	947	-	1159
12	Head height (HHt)	1052.6 ± 11.19	999	-	1087
13	Postmentum median length (PsMdL)	1014.9 ± 11.20	959	-	1083
14	Postmentum maximum width (PsMxW)	439.4 ± 12.62	329	-	466
15	Postmentum minimum width (PsMnW)	248.4 ± 3.17	227	-	258
16	Postmentum anterior width (PsAW)	241.5 ± 2.49	231	-	254
17	Postmentum seta (PsS)*	10.4 ± 0.88	6	-	15
18	Pronotum median length (PrMdL)	508.6 ± 4.66	484	-	531
19	Pronotum maximum length (PrMxL)	573.9 ± 5.97	548	-	600
20	Pronotum maximum width (PrMxW)	1024 ± 6.89	997	-	1069
21	Pronotum seta*(PrS)	62.9 ± 1.42	57	-	71
22	Pronotum margin seta (PrMS)***	40.6 ± 1.00	37	-	45
23	Pronotum disc seta (PrMS)***	22.3 ± 0.82	18	-	26
24	Mesonotum maximum width (MsMxW)	894.4 ± 5.64	872	-	926
25	Metanotum maximum width (MtMxW)	1023.5 ± 6.46	996	-	1066

	Characters (μm)	*C. elisae* (n = 10)			
		**Mean**	**Min.**		**Max.**
26	Hind femora length (HiFL)	1116.6 ± 9.96	1070	-	1171
27	Hind tibia length (HiTL)	1238.9 ± 4.87	1223	-	1265
28	Total body length without Head (TBLH)	2939.9 ± 94.99	2548	-	3362
29	Head - mandible width difference (ΔHMW)***	561.6 ± 33.03	408	-	795
30	Head length - head width (ΔHLHW)***	131.6 ± 19.19	25	-	214
31	Head index (HW/HLBM)***	0.9197 ± 0.01	0.88	-	0.98
32	Head contraction index (HW/HWBM)***	0.5669 ± 0.00	0.55	-	0.59
33	Mandible index (LML/HLBM)***	0.6551 ± 0.02	0.54	-	0.74
34	Postmentum index (PsNW/PsMW)***	0.5702 ± 0.02	0.5	-	0.74
35	Pronotum index (PrMW/HW)***	0.6876 ± 0.01	0.66	-	0.71
36	Mesonotum index (MsMW/PrMW)***	0.8735 ± 0.00	0.85	-	0.89
37	Metanotum index (MtMW/PrMW)***	0.9998 ± 0.01	0.97	-	1.03
