# Supplementary material for: Morphometric Analysis of Coptotermes spp. Soldier Caste (Blattodea: Rhinotermitidae) in Indonesia and Evidence of Coptotermes gestroi Extreme Head-Capsule Shapes
Source: Insects. 2021 May 20;12(5):477. doi: 10.3390/insects12050477 (PMC8160883; doi:10.3390/insects12050477)
Supplement: Supplementary file 1 [file insects-12-00477-s001.zip › Table S10.pdf]

Table S10. Standardized canonical discriminant matrix by DFA for *C. gestroi* with head morphology variation. Six components were extracted. Six sets of character were explained. Total % of Variance covered by the first two components was 64%.

**Sets of Character**	**Characters**	**Function**					
		1	2	3	4	5	6
**Head Capsule morph.**	Zscore(Log10HLM)	-0.469	0.331	0.455	0.314	0.474	-1.224
	Zscore(Log10HW)	0.018	2.512	0.256	-0.351	-1.732	0.739
**Labium features morph.**	Zscore(Log10HHt)	-1.312	-1.649	-0.126	0.174	0.278	1.184
	Zscore(Log10PsMxW)	0.204	0.365	-1.418	0.138	1.241	-0.145
	Zscore(Log10PsMnW)	0.916	-0.228	0.572	0.073	-0.586	-0.485
**Mandible morph.**	Zscore(Log10PsAW)	0.346	0.134	0.300	-0.590	0.624	0.510
**Leg morph.**	Zscore(Log10LbMxL)	0.418	-0.076	-0.507	0.786	-0.023	0.268
**Head Features morph.**	Zscore(Log10HiTL)	0.242	-1.145	1.020	0.253	0.378	-0.466
**Trunk morph.**	Zscore(Log10HLM)	-0.469	0.331	0.455	0.314	0.474	-1.224
**% of Variance**		35.7	28.3	19.5	8.9	6.9	0.7
