# Supplementary material for: Morphometric Analysis of Coptotermes spp. Soldier Caste (Blattodea: Rhinotermitidae) in Indonesia and Evidence of Coptotermes gestroi Extreme Head-Capsule Shapes
Source: Insects. 2021 May 20;12(5):477. doi: 10.3390/insects12050477 (PMC8160883; doi:10.3390/insects12050477)
Supplement: Supplementary file 1 [file insects-12-00477-s001.zip › Table S11.pdf]

Table S11. The amount of data variance covered by the four highest Canonical Variate produced from CVA of the whole species Head Capsule Shape. The number of iterations is 10,000.

**Component**	**Eigenvalues**	**% Variance**	**Cumulative %**
1.	17.24663818	50.063	50.063
2.	11.63894396	33.785	83.848
3.	4.05184165	11.761	95.609
4.	1.51264232	4.391	100.000
