# Supplementary material for: Morphometric Analysis of Coptotermes spp. Soldier Caste (Blattodea: Rhinotermitidae) in Indonesia and Evidence of Coptotermes gestroi Extreme Head-Capsule Shapes
Source: Insects. 2021 May 20;12(5):477. doi: 10.3390/insects12050477 (PMC8160883; doi:10.3390/insects12050477)
Supplement: Supplementary file 1 [file insects-12-00477-s001.zip › Table S12.pdf]

Table S12. Head Capsule Shape Classification results by DFA for the whole species. In Head Capsule shape analysis, comparisons were done in pairwise way (CC: *C. curvignathus*; CE: *C. elisae*; CG: *C. gestroi*; CK: *C. kashoveni*; CS: *C. sepangensis*).

**Species**		**Allocated to**				
		**CC**	**CE**	**CG**	**CK**	**CS**
**CC**		57	8	3	0	5
	%	100	14	5.3	0	8.8
**CE**		4	10	1	0	0
	%	40	100	10	0	0
**CG**		9	2	82	0	5
	%	11	2.4	100	0	6%
**CK**		0	0	0	5	2
	%	0	0	0	100	40
**CS**		7	0	3	2	56
	%	12.5	0	5.4	3.4	100
