# Supplementary material for: Morphometric Analysis of Coptotermes spp. Soldier Caste (Blattodea: Rhinotermitidae) in Indonesia and Evidence of Coptotermes gestroi Extreme Head-Capsule Shapes
Source: Insects. 2021 May 20;12(5):477. doi: 10.3390/insects12050477 (PMC8160883; doi:10.3390/insects12050477)
Supplement: Supplementary file 1 [file insects-12-00477-s001.zip › Table S13.pdf]

Table S13. The amount of data variance covered by three Canonical Variate produced from CVA of the whole species Postmentum Shape. The number of iterations is 10,000.

**Component**	**Eigenvalues**	**% Variance**	**Cumulative %**
1.	5.15907352	62.674	62.674
2.	2.56683534	31.183	93.857
3.	0.35532436	4.317	98.173
4.	0.15035768	1.827	100.000
