# Supplementary material for: Morphometric Analysis of Coptotermes spp. Soldier Caste (Blattodea: Rhinotermitidae) in Indonesia and Evidence of Coptotermes gestroi Extreme Head-Capsule Shapes
Source: Insects. 2021 May 20;12(5):477. doi: 10.3390/insects12050477 (PMC8160883; doi:10.3390/insects12050477)
Supplement: Supplementary file 1 [file insects-12-00477-s001.zip › Table S14.pdf]

Table S14. Postmentum Shape Classification results by DFA for the whole species. In Postmentum shape analysis, comparisons were done in pairwise way (CC: *C. curvignathus*; CE: *C. elisae*; CG: *C. gestroi*; CK: *C. kashoveni*; CS: *C. sepangensis*).

**Species**		**Allocated to**				
		**CC**	**CE**	**CG**	**CK**	**CS**
**CC**		65	8	0	0	0
	%	100	12.3	0	0	0
**CE**		0	9	0	0	0
	%	0	100	0	0	0
**CG**		4	2	80	1	0
	%	5	2.5	100	1.3	0
**CK**		0	0	1	3	2
	%	0	0	33	100	67
**CS**		2	1	3	2	58
	%	3.4	1.7	5.2	3.4	100
