# Supplementary material for: Morphometric Analysis of Coptotermes spp. Soldier Caste (Blattodea: Rhinotermitidae) in Indonesia and Evidence of Coptotermes gestroi Extreme Head-Capsule Shapes
Source: Insects. 2021 May 20;12(5):477. doi: 10.3390/insects12050477 (PMC8160883; doi:10.3390/insects12050477)
Supplement: Supplementary file 1 [file insects-12-00477-s001.zip › Table S15.pdf]

Table S15. The amount of data variance covered by the four highest Canonical Variate produced from CVA of the whole species Posmentum Lateral View. The number of iterations is 10,000.

Component	Eigenvalues	% Variance	Cumulative %
1.	6.70251598	65.816	65.816
2.	2.07432538	20.369	86.186
3.	0.93942409	9.225	95.410
4.	0.46739724	4.590	100.000
