# Supplementary material for: Morphometric Analysis of Coptotermes spp. Soldier Caste (Blattodea: Rhinotermitidae) in Indonesia and Evidence of Coptotermes gestroi Extreme Head-Capsule Shapes
Source: Insects. 2021 May 20;12(5):477. doi: 10.3390/insects12050477 (PMC8160883; doi:10.3390/insects12050477)
Supplement: Supplementary file 1 [file insects-12-00477-s001.zip › Table S16.pdf]

Table S16. Postmentum lateral view classification results by DFA for the whole species. In Head Capsule shape analysis, comparisons were done in pairwise way (CC: *C. curvignathus*; CE: *C. elisae*; CG: *C. gestroi*; CK: *C. kashoveni*; CS: *C. sepangensis*)..

Species		Allocated to				
		**CC**	**CE**	**CG**	**CK**	**CS**
**CC**		33	7	9	2	2
	%	100	21.2	27.3	6.1	6.1
**CE**		0	4	1	0	1
	%	8	100	25	0	25
**CG**		12.7	1	82	0	3
	%	11	1.6	100	0	4.8%
**CK**		1	1	0	5	3
	%	25	25	0	100	75
**CS**		3	0	3	4	56
	%	9.1	0	9.1	12.1	100
