# Supplementary material for: Morphometric Analysis of Coptotermes spp. Soldier Caste (Blattodea: Rhinotermitidae) in Indonesia and Evidence of Coptotermes gestroi Extreme Head-Capsule Shapes
Source: Insects. 2021 May 20;12(5):477. doi: 10.3390/insects12050477 (PMC8160883; doi:10.3390/insects12050477)
Supplement: Supplementary file 1 [file insects-12-00477-s001.zip › Table S17 & S18.pdf]

Table S17. Pairwise distance matrix (uncorrected p-distances %) for all the 12S sequence used in this study.

	Samples	1 23*	2 24*	3 25*	4 26*	5 27*	6 28*	7 29*	8 30*	9	10	11	12	13	14	15	16	17	18	19	20	21	22
1	CPCG001INA_C._gestroi_Cipinang																						
2	PKCG002INA_C._gestroi_Pondokkelapa	0.000																					
3	GSCG001INA_C._gestroi_Gunungsindur	0.005	0.005																				
4	CICG001INA_C._gestroi_Cibinong	0.005	0.005	0.000																			
5	BTCG001INA_C._gestroi_Batam	0.007	0.007	0.002	0.002																		
6	KRCG001INA_C._gestroi_Karadenan	0.005	0.005	0.000	0.000	0.002																	
7	PPCG001INA_C._gestroi_Parungpanjang	0.005	0.005	0.000	0.000	0.002	0.000																
8	BTCG002INA_C._gestroi_Batam	0.002	0.002	0.002	0.002	0.005	0.002	0.002															
9	KU925205.1_C._gestroi	0.005	0.005	0.005	0.005	0.007	0.005	0.005	0.002														
10	KU925208.1_C._heimi	0.017	0.017	0.017	0.017	0.020	0.017	0.017	0.015	0.017													
11	KU925206.1_C._heimi	0.012	0.012	0.012	0.012	0.015	0.012	0.012	0.010	0.012	0.005												
12	SICC001INA_C._curvignathus_Simeulue	0.032	0.032	0.032	0.032	0.034	0.032	0.032	0.029	0.027	0.034	0.029											
13	KBCC001INA_C._curvignathus_Karimunbesar	0.037	0.037	0.037	0.037	0.039	0.037	0.037	0.034	0.032	0.039	0.034	0.005										
14	PPCC001INA_C._curvignathus_Parungpanjang	0.037	0.037	0.037	0.037	0.039	0.037	0.037	0.034	0.032	0.039	0.034	0.005	0.000									
15	ASCC003INA_C._elisae_Asmat	0.034	0.034	0.034	0.034	0.037	0.034	0.034	0.032	0.029	0.037	0.032	0.002	0.002	0.002								
16	KU925201.1_C._elisae	0.032	0.032	0.032	0.032	0.034	0.032	0.032	0.029	0.027	0.034	0.029	0.005	0.005	0.005	0.002							
17	SICS001INA_C._sepangensis_Simeulue	0.025	0.025	0.020	0.020	0.022	0.020	0.020	0.022	0.025	0.027	0.022	0.029	0.029	0.029	0.027	0.029						
18	BTCS001INA_C._sepangensis_Batam	0.025	0.025	0.020	0.020	0.022	0.020	0.020	0.022	0.025	0.027	0.022	0.029	0.029	0.029	0.027	0.029	0.000					
19	KU925215.1_C._sepangensis	0.025	0.025	0.020	0.020	0.022	0.020	0.020	0.022	0.025	0.027	0.022	0.029	0.029	0.029	0.027	0.029	0.000	0.000				
20	PPCK001INA_C._kalshoveni_Parungpanjang	0.032	0.032	0.032	0.032	0.034	0.032	0.032	0.029	0.032	0.034	0.029	0.029	0.029	0.029	0.027	0.029	0.015	0.015	0.015			
21	KU925210.1_C._kalshoveni	0.032	0.032	0.032	0.032	0.034	0.032	0.032	0.029	0.032	0.034	0.029	0.037	0.037	0.037	0.034	0.037	0.012	0.012	0.012	0.007		
22	KU925209.1_C._kalshoveni	0.032	0.032	0.032	0.032	0.034	0.032	0.032	0.029	0.032	0.034	0.029	0.037	0.037	0.037	0.034	0.037	0.012	0.012	0.012	0.007	0.000	
23	WYCF001JP_C._formosanus_Wakayama	0.027	0.027	0.027	0.027	0.029	0.027	0.027	0.025	0.027	0.034	0.029	0.037	0.037	0.037	0.034	0.037	0.022	0.022	0.022	0.022	0.025	0.025
24	AB626145.1_C._formosanus	0.027	0.027	0.027	0.027	0.029	0.027	0.027	0.025	0.027	0.034	0.029	0.037	0.037	0.037	0.034	0.037	0.022	0.022	0.022	0.022	0.025	0.025
25	AB626147.1_C._formosanus	0.027	0.027	0.027	0.027	0.029	0.027	0.027	0.025	0.027	0.034	0.029	0.037	0.037	0.037	0.034	0.037	0.022	0.022	0.022	0.022	0.025	0.025
26	KU925227.1_Heterotermes_malabaricus	0.037	0.037	0.037	0.037	0.039	0.037	0.037	0.034	0.037	0.039	0.034	0.049	0.054	0.054	0.051	0.049	0.032	0.032	0.032	0.034	0.029	0.029
27	KU925235.1_Heterotermes_validus	0.042	0.042	0.042	0.042	0.044	0.042	0.042	0.039	0.042	0.039	0.034	0.049	0.054	0.054	0.051	0.054	0.032	0.032	0.032	0.034	0.034	0.034
28	KP026256.1_Prorhinotermes_canalifrons	0.103	0.103	0.108	0.108	0.110	0.108	0.108	0.105	0.108	0.103	0.108	0.120	0.125	0.125	0.123	0.123	0.110	0.110	0.110	0.115	0.115	0.115
29	MK541931.1_Reticulitermes_leptomandibularis	0.074	0.074	0.069	0.069	0.071	0.069	0.069	0.071	0.069	0.076	0.074	0.078	0.083	0.083	0.081	0.081	0.071	0.071	0.071	0.078	0.083	0.083
30	CIOJ001INA_Odontotermes_sp.	0.093	0.093	0.093	0.093	0.096	0.093	0.093	0.091	0.093	0.091	0.088	0.103	0.108	0.108	0.105	0.105	0.091	0.091	0.091	0.096	0.098	0.098
24*	AB626145.1_C._formosanus	0.000																					
25*	AB626147.1_C._formosanus	0.000	0.000																				
26*	KU925227.1_Heterotermes_malabaricus	0.032	0.032	0.032																			
27*	KU925235.1_Heterotermes_validus	0.037	0.037	0.037	0.039																		
28*	KP026256.1_Prorhinotermes_canalifrons	0.098	0.098	0.098	0.115	0.108																	
29*	MK541931.1_Reticulitermes_leptomandibularis	0.074	0.074	0.074	0.086	0.078	0.120																
30*	CIOJ001INA_Odontotermes_sp.	0.088	0.088	0.088	0.096	0.083	0.135	0.105															

Table S18. Pairwise distance matrix (uncorrected p-distances %) for all the 16S sequence used in this study.

	Samples	1 23*	2 24*	3 25*	4 26*	5 27*	6 28*	7 29*	8 30*	9	10	11	12	13	14	15	16	17	18	19	20	21	22
1	CPCG001INA_C._gestroi_Cipinang																						
2	PKCG002INA_C._gestroi_Pondokkelapa	0.002																					
3	GSCG001INA_C._gestroi_Gunungsindur	0.002	0.000																				
4	CICG001INA_C._gestroi_Cibinong	0.002	0.000	0.000																			
5	BTCG001INA_C._gestroi_Batam	0.002	0.000	0.000	0.000																		
6	KRCG001INA_C._gestroi_Karadenan	0.002	0.000	0.000	0.000	0.000																	
7	PPCG001INA_C._gestroi_Parungpanjang	0.008	0.007	0.007	0.007	0.007	0.007																
8	BTCG002INA_C._gestroi_Batam	0.002	0.003	0.003	0.003	0.003	0.003	0.010															
9	KU925205.1_C._gestroi	0.007	0.005	0.005	0.005	0.005	0.005	0.002	0.008														
10	KU925208.1_C._heimi	0.008	0.007	0.007	0.007	0.007	0.007	0.013	0.010	0.012													
11	KU925206.1_C._heimi	0.008	0.007	0.007	0.007	0.007	0.007	0.013	0.010	0.012	0.000												
12	SICC001INA_C._curvignathus_Simeulue	0.034	0.032	0.032	0.032	0.032	0.032	0.032	0.035	0.030	0.025	0.025											
13	KBCC001INA_C._curvignathus_Karimunbesar	0.034	0.032	0.032	0.032	0.032	0.032	0.032	0.035	0.030	0.025	0.025	0.000										
14	PPCC001INA_C._curvignathus_Parungpanjang	0.034	0.032	0.032	0.032	0.032	0.032	0.032	0.035	0.030	0.025	0.025	0.000	0.000									
15	ASCC003INA_C._elisae_Asmat	0.037	0.035	0.035	0.035	0.035	0.035	0.035	0.039	0.034	0.029	0.029	0.005	0.005	0.005								
16	KU925201.1_C._elisae	0.037	0.035	0.035	0.035	0.035	0.035	0.035	0.039	0.034	0.029	0.029	0.005	0.005	0.005	0.000							
17	SICS001INA_C._sepangensis_Simeulue	0.042	0.040	0.040	0.040	0.040	0.040	0.039	0.044	0.037	0.040	0.040	0.042	0.042	0.042	0.042	0.042						
18	BTCS001INA_C._sepangensis_Batam	0.044	0.042	0.042	0.042	0.042	0.042	0.040	0.045	0.039	0.042	0.042	0.040	0.040	0.040	0.040	0.040	0.005					
19	KU925215.1_C._sepangensis	0.037	0.035	0.035	0.035	0.035	0.035	0.035	0.039	0.034	0.035	0.035	0.037	0.037	0.037	0.037	0.037	0.008	0.010				
20	PPCK001INA_C._kalshoveni_Parungpanjang	0.037	0.035	0.035	0.035	0.035	0.035	0.035	0.039	0.034	0.032	0.032	0.029	0.029	0.029	0.029	0.029	0.027	0.029	0.022			
21	KU925210.1_C._kalshoveni	0.039	0.037	0.037	0.037	0.037	0.037	0.034	0.040	0.032	0.037	0.037	0.034	0.034	0.034	0.034	0.034	0.027	0.029	0.023	0.005		
22	KU925209.1_C._kalshoveni	0.039	0.037	0.037	0.037	0.037	0.037	0.034	0.040	0.032	0.037	0.037	0.034	0.034	0.034	0.034	0.034	0.027	0.029	0.023	0.008	0.003	
23	WYCF001JP_C._formosanus_Wakayama	0.042	0.040	0.040	0.040	0.040	0.040	0.039	0.044	0.037	0.039	0.039	0.042	0.042	0.042	0.040	0.040	0.045	0.044	0.044	0.047	0.050	0.050
24	AB626145.1_C._formosanus	0.042	0.040	0.040	0.040	0.040	0.040	0.039	0.044	0.037	0.039	0.039	0.042	0.042	0.042	0.040	0.040	0.045	0.044	0.044	0.047	0.050	0.050
25	AB626147.1_C._formosanus	0.042	0.040	0.040	0.040	0.040	0.040	0.039	0.044	0.037	0.039	0.039	0.042	0.042	0.042	0.040	0.040	0.045	0.044	0.044	0.047	0.050	0.050
26	KU925227.1_Heterotermes_malabaricus	0.055	0.054	0.054	0.054	0.054	0.054	0.054	0.057	0.052	0.054	0.054	0.049	0.049	0.049	0.049	0.049	0.054	0.052	0.049	0.049	0.047	0.047
27	KU925235.1_Heterotermes_validus	0.065	0.064	0.064	0.064	0.064	0.064	0.062	0.067	0.060	0.067	0.067	0.060	0.060	0.060	0.060	0.060	0.059	0.057	0.057	0.064	0.064	0.064
28	KP026256.1_Prorhinotermes_canalifrons	0.131	0.129	0.129	0.129	0.129	0.129	0.131	0.131	0.129	0.131	0.131	0.126	0.126	0.126	0.124	0.124	0.136	0.131	0.128	0.126	0.128	0.126
29	MK541931.1_Reticulitermes_leptomandibularis	0.106	0.104	0.104	0.104	0.104	0.104	0.107	0.107	0.106	0.102	0.102	0.099	0.099	0.099	0.104	0.104	0.102	0.101	0.101	0.107	0.111	0.109
30	CIOJ001INA_Odontotermes_sp.	0.104	0.102	0.102	0.102	0.102	0.102	0.106	0.106	0.104	0.106	0.106	0.107	0.107	0.107	0.107	0.107	0.114	0.112	0.107	0.111	0.111	0.112
24*	AB626145.1_C._formosanus	0.000																					
25*	AB626147.1_C._formosanus	0.000	0.000																				
26*	KU925227.1_Heterotermes_malabaricus	0.059	0.059	0.059																			
27*	KU925235.1_Heterotermes_validus	0.049	0.049	0.049	0.067																		
28*	KP026256.1_Prorhinotermes_canalifrons	0.126	0.126	0.126	0.139	0.136																	
29*	MK541931.1_Reticulitermes_leptomandibularis	0.104	0.104	0.104	0.111	0.116	0.154																
30*	CIOJ001INA_Odontotermes_sp.	0.111	0.111	0.111	0.116	0.122	0.153	0.131															
