# Supplementary material for: Morphometric Analysis of Coptotermes spp. Soldier Caste (Blattodea: Rhinotermitidae) in Indonesia and Evidence of Coptotermes gestroi Extreme Head-Capsule Shapes
Source: Insects. 2021 May 20;12(5):477. doi: 10.3390/insects12050477 (PMC8160883; doi:10.3390/insects12050477)
Supplement: Supplementary file 1 [file insects-12-00477-s001.zip › Table S2.pdf]

Table S2. Rotated Component Matrix by Varimax. Two components and seven sets of character were explained. Total % of Variance covered 82.43%.

Sets of Character	Characters	Component	
		1	2
**Head Capsule Morphometric**	Zscore(Log10HWBM)	**0.932**	0.242
	Zscore(Log10HLM)	0.922	0.276
	Zscore(Log10HLBM)	0.896	0.377
	Zscore(Log10HW)	0.848	0.497
	Zscore(Log10BML)	0.845	0.130
	Zscore(Log10HHt)	0.832	0.458
**Labium Morphometric**	Zscore(Log10PsMxW)	0.918	0.176
	Zscore(Log10PsMdL)	0.898	-0.013
	Zscore(Log10PsAW)	0.840	0.150
**Trunk Morphometric**	Zscore(Log10PrMxW)	0.875	0.438
	Zscore(Log10PrMdL)	0.864	0.426
	Zscore(Log10PrMxL)	0.829	0.494
	Zscore(Log10MsMxW)	0.800	0.528
	Zscore(Log10MtMxW)	0.793	0.485
	Zscore(Log10TBLH)	0.766	-0.159
**Head Features Morphometric**	Zscore(Log10LML)	0.866	0.375
	Zscore(Log10LbMxL)	0.848	-0.003
	Zscore(Log10FW)	0.618	0.579
	Zscore(Log10LbMxW)	0.594	0.137
**Leg Morphometric**	Zscore(Log10HiFL)	0.812	0.482
	Zscore(Log10HiTL)	0.802	0.520
**Seta Number**	Zscore(Log10PrS)	0.208	**0.921**
	Zscore(Log10HCS)	0.228	0.860
	Zscore(Log10PsS)	-0.119	0.752
**% of Variance**		61.13	21.3
