# Supplementary material for: Morphometric Analysis of Coptotermes spp. Soldier Caste (Blattodea: Rhinotermitidae) in Indonesia and Evidence of Coptotermes gestroi Extreme Head-Capsule Shapes
Source: Insects. 2021 May 20;12(5):477. doi: 10.3390/insects12050477 (PMC8160883; doi:10.3390/insects12050477)
Supplement: Supplementary file 1 [file insects-12-00477-s001.zip › Table S3.pdf]

Table S3. Characteristics comparison of *Coptotermes* spp.

Species	Characteristics						
	Dorsal View						
	**Head Capsule**	**Clypeus**	**Labrum**	**Mandible**	**Anennae**	**Pronotum**	**Abdomen**
C. curvignathus	subangular-shaped, posteriorly bulged, Considerable numbers on HC (max. 56)	trapezoid-shape	broad tongue-shaped with hyaline tip. One pair of long terminal setae and one pair of medium-length para-terminal setae (2+2M labrum seta formula).	Strongly Incurved, no lateral teeth.	15 – 18 segments. In one specimen, a right antenna with 18 segments was paired with a left antenna with 16 segments. The antenna formula varied, with the 4th and 3rd segments shorter compared to the 2nd and 5th segments.	Flat, with a reversed trapezoid shape, and had a considerable number of up to 113 setae on the disc and at the edge. The anterior margin of the pronotum was highly indented, but less indented on the posterior part. The anterior corner margins were roundly angled, while the posterior corner margins were roundly wide-angled. A median complexion appeared from the pronotum to the most posterior segment.	At the abdominal sternite and tergite, the setae were situated randomly and sparsely with at least one line of setae on the posterior edge of each segment. The setae were shorter in the sternite part.
C. elisae	subangular-shaped, posteriorly bulged, Considerable numbers on HC (max. 48)	trapezoid-shape	Broad tongue-shaped with hyaline tip. One pair of long terminal setae and one pair of medium-length para-terminal setae (2+2M labrum seta formula).	Strongly Incurved, no lateral teeth.	15 - 16 segments. The antenna formula varied, with the 4th and 3rd segments shorter compared to the 2nd and 5th segments.	Flat, with a reversed trapezoid shape, and had a considerable number of up to 71 setae on the disc and at the edge. The anterior margin of the pronotum was highly indented, but less indented on the posterior part. The anterior corner margins were roundly angled, while the posterior corner margins were roundly wide-angled. A median complexion appeared from the pronotum to the most posterior segment.	At the abdominal sternite and tergite, the setae were situated randomly and sparsely with at least one line of setae on the posterior edge of each segment. The setae were shorter in the sternite part.
C. gestroi	Three extreme HC: (1) Several specimens had broadly oval head-shape, regardless of their size, the head looked similar to the head of C. curvignathus from the dorsal view at first glance; (2) few specimens had subangular head-shape (Figure 2); while (3) majority specimens had the usual egg-shaped. Few setae on the HC (max. 23).	trapezoid-shape	Narrow tongue-shaped with hyaline tip. it had one pair of setae and one pair of short para-terminal setae (2+2S labrum seta formula). In some specimens, the existence of para-terminal setae was either not obvious or not existed.	Slightly incurved, rather straight, and the inner side of the mandible was less concave. No lateral teeth.	13 - 16 segments. Generally, the 3rd segment was the shortest and the 5th segment was the longest in a comparison of the 2nd – 5th segments.	Flat with setae mostly found at the edge of the pronotum. Few or no setae were observed on the disc part of the pronotum (max. 5). The anterior margin was strongly indented while the posterior was weak. The anterior corner margin was rounded and narrowly angled while the posterior corner was rounded and widely angled. A median complexion appeared from the pronotum to the most posterior segment.	The setae at the abdominal sternite and tergite part were positioned randomly and sparsely with at least one line of setae on the posterior edge of each segment. The setae were shorter in the sternite part.

Table S3. Characteristics comparison of *Coptotermes* spp. (Continued)

Species	Characteristics						
	Dorsal View						
	**Head Capsule**	**Clypeus**	**Labrum**	**Mandible**	**Anennae**	**Pronotum**	**Abdomen**
C. sepangensis	subangular-shaped, narrow at the anterior and rounded at the posterior part. moderate number of setae decorated the HC (max. 32).	trapezoid-shape	Narrow tongue-shaped. one pair of terminal setae and one pair of medium-length para-terminal setae (2+2M labrum seta formula).	Quite incurved with its curvature starting at the early part of the mandible. The level of curvature varied among specimens.	13 - 15 segments. Mostly, the 3rd or 4th segment was the shortest and the 2nd segment was longer. The 5th segment was the longest in a comparison among the 2nd -5th segments	Flat with a moderate number of scattered setae on the edge and the disc. The pronotum was reverse-bun-shaped. The anterior margin was well indented, but the indentation was less at the posterior margin. The anterior corner margin was rounded at a narrowed angle while the posterior corner margin was rounded at a wider angle.	Abdominal sternite setae were very short and scattered setae on its surface. Abdominal tergite setae on the posterior margin of the segment was less obvious
C. kalshoveni	generally broad at the middle with a sudden narrowing at the anterior part just at the posterior of the mandible. A massive number of setae was observed on the HC (max. 53).	trapezoid-shape	Narrow tongue-shaped. The labrum terminal had one pair of terminals and one pair of short para-terminal setae (2+2S labrum seta formula). Some specimens had para-terminal setae situated close to the labrum margin rather than exactly on the back of the terminal setae.	Less incurved, with the curvature starting at the final third of the distal part.	15 segments. The 2nd segment was the longest segment among the first four segments, excluding the scape part (3<4<5<2).	Flat and had a high number of setae scattered on the pronotum disc and marginal side. The pronotum anterior margin was well indented, while the posterior margin was less indented. The anterior corner margin was narrowly rounded while the posterior corner margin was widely rounded.	Abdominal sternite had scattered setae on its surface. Abdominal tergite's row of setae on the posterior margin of the segment was less obvious.

Table S3. Characteristics comparison of *Coptotermes* spp. (Continued)

Species	Characteristics			
	Lateral View		Ventral View	Anterior View
	Head Capsule	Antennae	Postmentum	Fontanelle
C. curvignathus	Rounded-shaped, narrowed anteriorly. Non prominent frons. Setae positioned diagonally from the posterior part of the HC anteriorly down to the gena.	Antennal socket protruded dorsally.	Low waist PS was observed with diverse numbers of setae ranging up to 18 at the anterior edge.	subangular-shaped, generally with one pair of setae. However, 1 - 4 number of setae were observed.
C. elisae	Rounded-shaped, narrowed anteriorly. Non prominent frons. Setae positioned diagonally from the posterior part of the HC anteriorly down to the gena.	Antennal socket protruded dorsally.	Low waist PS was observed with diverse numbers of setae ranging up to 15 at the anterior edge.	subangular-shaped, with one pair of setae.
C. gestroi	Anteriorly narrowed. The degree of prominence varied within colony.	Antennal socket protruded dorsally.	Middle waist PS was observed across the samples. Up to 15 setae on the postmentum.	asymmetrical oval-shaped . Generally, with a pair of setae. However, variations occurred in which a few individuals in some studied populations had 3 – 4 setae around the fontanelle.
C. sepangensis	PS bulged ventrally. Moderate numbers of setae were observed. The frons slightly bulged.	Antennal socket protruded dorsally.	Low-waist PS was observed. Up to 15 setae were located at the anterior part of the PS.	subangular-shaped, with generally one pair of setae. A few individuals in the study showed three setae around the fontanelle.
C. kalshoveni	Broad HC. The PS bulged. A considerable number of setae were observed from the lateral view. The frons bulged and the fontanelle opening was relatively perpendicular with the clypeus.	Antennal socket protruded dorsally.	Low-waist PS with a Up to 13 setae at the anterior part of PS. Within *Coptotermes* spp. collected in this study, *C. kalshoveni* has waist width wider than the anterior margin width.	asymmetrical oval-shaped . Two pairs of setae were situated around the fontanelle.
