# Supplementary material for: Morphometric Analysis of Coptotermes spp. Soldier Caste (Blattodea: Rhinotermitidae) in Indonesia and Evidence of Coptotermes gestroi Extreme Head-Capsule Shapes
Source: Insects. 2021 May 20;12(5):477. doi: 10.3390/insects12050477 (PMC8160883; doi:10.3390/insects12050477)
Supplement: Supplementary file 1 [file insects-12-00477-s001.zip › Table S4.pdf]

Table S4. Collinearity diagnostics conducted by Linear Regression analysis. Tolerance value < 0.1 or VIF > 10.00 explained collinearity. Head width character was treated as dependent variable.

**Characters**	**Collinearity Statistics**	
	**Tolerance**	**VIF**
Zscore(Log10HLM)	0.071	14.020
Zscore(Log10HLBM)	0.048	21.046
Zscore(Log10HWBM)	0.054	18.389
Zscore(Log10LML)	0.081	12.347
Zscore(Log10HHt)	0.078	12.763
Zscore(Log10PsMxW)	0.143	7.014
Zscore(Log10PrMdL)	0.015	66.422
Zscore(Log10PrMxL)	0.013	77.871
Zscore(Log10PrMxW)	0.015	68.208
Zscore(Log10MsMxW)	0.019	51.326
Zscore(Log10MtMxW)	0.042	23.588
Zscore(Log10HiFL)	0.088	11.364
Zscore(Log10HiTL)	0.065	15.375

Dependent Variable: Zscore(Log10HW)
