# Supplementary material for: Morphometric Analysis of Coptotermes spp. Soldier Caste (Blattodea: Rhinotermitidae) in Indonesia and Evidence of Coptotermes gestroi Extreme Head-Capsule Shapes
Source: Insects. 2021 May 20;12(5):477. doi: 10.3390/insects12050477 (PMC8160883; doi:10.3390/insects12050477)
Supplement: Supplementary file 1 [file insects-12-00477-s001.zip › Table S5.pdf]

Table S5. Collinearity diagnostics conducted by Linear Regression analysis. Tolerance value < 0.1 or VIF > 10.00 explained collinearity. Head width character was treated as dependent variable.

**Characters**	**Collinearity Statistics**	
	**Tolerance**	**VIF**
Zscore(Log10PsMxW)	**0.172**	**5.817**
Zscore(Log10PrMdL)	0.035	28.490
Zscore(Log10PrMxL)	0.023	42.877
Zscore(Log10PrMxW)	0.016	61.696
Zscore(Log10MsMxW)	0.024	40.831
Zscore(Log10MtMxW)	0.048	20.736

Dependent Variable: Head Width
