# Supplementary material for: Morphometric Analysis of Coptotermes spp. Soldier Caste (Blattodea: Rhinotermitidae) in Indonesia and Evidence of Coptotermes gestroi Extreme Head-Capsule Shapes
Source: Insects. 2021 May 20;12(5):477. doi: 10.3390/insects12050477 (PMC8160883; doi:10.3390/insects12050477)
Supplement: Supplementary file 1 [file insects-12-00477-s001.zip › Table S6.pdf]

Table S6. Rotated Component Matrix by Varimax. Two components were extracted. Five sets of character were explained. Total % of Variance covered were 76.79%.

Sets of Character	Characters	Component	
		1	2
Head Capsule Morphometric	Zscore(Log10PrMxW)	0.979	-0.041
	Zscore(Log10PrMxL)	0.967	-0.030
	Zscore(Log10MsMxW)	0.964	-0.068
	Zscore(Log10PrMdL)	0.956	-0.005
	Zscore(Log10MtMxW)	0.937	-0.053
Labium Morphometric	Zscore(Log10HW)	0.966	-0.031
	Zscore(Log10HHt)	0.896	-0.141
	Zscore(Log10HLM)	0.892	-0.344
	Zscore(Log10HWBM)	0.884	-0.072
	Zscore(Log10HLBM)	0.883	-0.041
	Zscore(Log10BML)	0.505	-0.656
Trunk Morphometric	Zscore(Log10PsMxW)	0.915	0.061
	Zscore(Log10PsMnW)	0.779	0.414
	Zscore(Log10PsAW)	0.615	0.373
Head Features Morphometric	Zscore(Log10LbMxW)	0.822	0.104
	Zscore(Log10LML)	0.784	-0.121
	Zscore(Log10LbMxL)	0.478	0.556
Leg Morphometric	Zscore(Log10HiTL)	0.810	0.266
	Zscore(Log10HiFL)	0.770	0.157
Seta Number	Zscore(Log10PrS)	-0.664	0.175
% of Variance		69.98	6.81
