# Supplementary material for: Morphometric Analysis of Coptotermes spp. Soldier Caste (Blattodea: Rhinotermitidae) in Indonesia and Evidence of Coptotermes gestroi Extreme Head-Capsule Shapes
Source: Insects. 2021 May 20;12(5):477. doi: 10.3390/insects12050477 (PMC8160883; doi:10.3390/insects12050477)
Supplement: Supplementary file 1 [file insects-12-00477-s001.zip › Table S7.pdf]

Table S7**.** The classification results obtained by discriminant function analysis for all of the species. Original and cross-validated classifications were included. Overall, 99.5% of groupings were solved by 11 variables. Cc: *C. curvignathus*; Cg: *C. gestroi*; Cs: *C. sepangensis*; Ce*: C. elisae*.

**Species**		**Predicted Group Membership**				**Total**
		**Cc**	**Cg**	**Cs**	**Ce**	
Cc		62	0	0	4	66
	%	93.9	0	0	6.1	100
Cg		0	83	1	0	83
	%	0	98.8	1.2	0	100
Cs		0	0	63	0	63
	%	0	0	100	0	100
Ce		0	0	0	10	10
	%	0	0	0	100	100
