# Supplementary material for: Morphometric Analysis of Coptotermes spp. Soldier Caste (Blattodea: Rhinotermitidae) in Indonesia and Evidence of Coptotermes gestroi Extreme Head-Capsule Shapes
Source: Insects. 2021 May 20;12(5):477. doi: 10.3390/insects12050477 (PMC8160883; doi:10.3390/insects12050477)
Supplement: Supplementary file 1 [file insects-12-00477-s001.zip › Table S8.pdf]

Table S8. Standardized canonical discriminant matrix of whole species DFA. Two components were extracted. Five sets of character were explained. Total % of Variance covered by two components was 100%.

Sets of Character	Characters	Function 1	Function 2
**Head Capsule morph.**	Zscore(Log10HW)	**0.903**	-0.286
**Seta Number**	Zscore(Log10PrS)	**0.822**	-0.216
	Zscore(Log10HCS)	0.471	0.124
	Zscore(Log10PsS)	-0.162	-0.148
**Labium morph.**	Zscore(Log10PsMdL)	-0.029	**0.897**
	Zscore(Log10PsAW)	-0.027	0.272
**Head Features morph.**	Zscore(Log10FW)	0.059	-0.395
	Zscore(Log10LbMxL)	0.117	0.489
**Trunk morph.**	Zscore(Log10TBLH)	-0.396	0.173
**% of Variance**		80.6	19.4
