# Supplementary material for: Morphometric Analysis of Coptotermes spp. Soldier Caste (Blattodea: Rhinotermitidae) in Indonesia and Evidence of Coptotermes gestroi Extreme Head-Capsule Shapes
Source: Insects. 2021 May 20;12(5):477. doi: 10.3390/insects12050477 (PMC8160883; doi:10.3390/insects12050477)
Supplement: Supplementary file 1 [file insects-12-00477-s001.zip › Table S9.pdf]

Table S9. The classification results obtained by discriminant function analysis for *C. gestroi* species with head morphology variation. Original and cross-validated classifications were included. Overall, 82.1% of groupings were step-wisely solved by engaging nine variables. CP: Cipinang; GS: Gunung Sindur; PP: Parung Panjang; CI: Cibinong; KR: Karadenan; BT: Batam; PK: Pondok Kelapa.

**Location**		**Predicted Group Membership**							**Total**
		CP	GS	PP	CI	KR	BT	PK	
CP		10	0	0	0	0	0	0	10
	%	100	0	0	0	0	0	0	100
GS		0	9	0	1	0	0	0	10
	%	0	90	0	10	0	0	0	100
PP		0	0	8	1	0	1	0	10
	%	0	0	80	10	0	10	0	100
CI		0	0	1	15	3	1	0	20
	%	0	0	5	75	15	5	0	100
KR		0	0	0	1	9	0	0	10
	%	0	0	0	10	90	0	0	100
BT		1	1	2	0	1	9	0	14
	%	7.1	7.1	14.3	0	7.1	64	0	100
PK		0	0	0	1	0	0	9	10
	%	0	0	0	10	0	0	90	100
